# Rice ubiquitin‐conjugating enzyme OsUbc13 negatively regulates immunity against pathogens by enhancing the activity of OsSnRK1a

**DOI:** 10.1111/pbi.14059

**Published:** 2023-04-27

**Authors:** Jianping Liu, Bo Nie, Boling Yu, Feiyun Xu, Qian Zhang, Ya Wang, Weifeng Xu

**Affiliations:** ^1^ Center for Plant Water‐use and Nutrition Regulation and College of Resources and Environment, Joint International Research Laboratory of Water and Nutrient in Crop Fujian Agriculture and Forestry University Fuzhou China; ^2^ College of Life Sciences Fujian Agriculture and Forestry University Fuzhou China; ^3^ Cereal Crops Research Institute, Henan Academy of Agricultural Sciences Zhengzhou China

**Keywords:** rice, OsUbc13, K63‐linked polyubiquitination, OsSnRK1a, pathogen resistance

## Abstract

Ubc13 is required for Lys63‐linked polyubiquitination and innate immune responses in mammals, but its functions in plant immunity still remain largely unknown. Here, we used molecular biological, pathological, biochemical, and genetic approaches to evaluate the roles of rice OsUbc13 in response to pathogens. The *OsUbc13*‐RNA interference (RNAi) lines with lesion mimic phenotypes displayed a significant increase in the accumulation of flg22‐ and chitin‐induced reactive oxygen species, and in defence‐related genes expression or hormones as well as resistance to *Magnaporthe oryzae* and *Xanthomonas oryzae* pv *oryzae*. Strikingly, OsUbc13 directly interacts with OsSnRK1a, which is the α catalytic subunit of SnRK1 (sucrose non‐fermenting‐1‐related protein kinase‐1) and acts as a positive regulator of broad‐spectrum disease resistance in rice. In the *OsUbc13*‐RNAi plants, although the protein level of OsSnRK1a did not change, its activity and ABA sensitivity were obviously enhanced, and the K63‐linked polyubiquitination was weaker than that of wild‐type Dongjin (DJ). Overexpression of the deubiquitinase‐encoding gene *OsOTUB1.1* produced similar effects with inhibition of *OsUbc13* in affecting immunity responses, *M*. *oryzae* resistance, OsSnRK1a ubiquitination, and OsSnRK1a activity. Furthermore, re‐interfering with *OsSnRK1a* in one *OsUbc13*‐RNAi line (Ri‐3) partially restored its *M*. *oryzae* resistance to a level between those of Ri‐3 and DJ. Our data demonstrate OsUbc13 negatively regulates immunity against pathogens by enhancing the activity of OsSnRK1a.

## Abstract 2

Inhibition of *OsUbc13* activates autoimmune responses, and enhances the activity of OsSnRK1a, one OsUbc13's direct interaction partners, through attenuating its K63‐linked polyubiquitination, thereby increasing pathogen resistance in rice.

## Introduction

Plants have evolved a two‐layered immune system to prevent the attack of pathogen: PAMP‐triggered immunity (PTI) governed by pattern recognition receptors and effector‐triggered immunity (ETI) conferred by highly polymorphic resistance (R) proteins (Faulkner and Robatzek, 2012; Jones and Dangl, 2006; Schwessinger and Zipfel, 2008; Spoel and Dong, 2012). One typical feature of ETI is the hypersensitive response (HR), which is an important defence mechanism employed by plants to protect themselves from biotic stress. In the infection site, plants rapidly undergo programmed cell death (PCD) that arrests the growth and spread of the pathogen (Wu *et al*., 2014; Zebell and Dong, 2015). HR is also usually accompanied by the generation of reactive oxygen species (ROS), expression of pathogenesis‐related (PR) proteins, accumulation of callose and thickening of cell walls at the infection sites (Jones and Dangl, 1996). Given its severe impact on cell fate and its high efficiency in fighting pathogen damage, HR must be tightly monitored in the absence of pathogen attack to balance plant growth and defence (Mukhtar *et al*., 2016).

Since plants lack adaptive immunity, their immune system is largely dependent on two types of innate immunity, PTI and ETI. Mammalian innate immunity is closely regulated by a cascade of ubiquitination systems (Jiang and Chen, 2011). Ubiquitination is a post‐translational protein modification that regulates numerous important cellular processes in all eukaryotes. Ubiquitin (Ub), a highly conserved 76‐amino acid polypeptide, is first activated by forming a thioester complex with the Ub‐activating enzyme (Uba or E1). Next, Ub‐conjugating enzyme (Ubc or E2) catalyses the transfer of activated Ub to Ub ligase (E3) depending on the active‐site cysteine. Finally, E3 mediates ubiquitination of the target proteins (Ye and Rape, 2009). The final fates of target proteins after ubiquitination usually depend on whether the protein is polyubiquitinated or monoubiquitinated. During polyubiquitination, the C‐terminal of the next Ub is connected to one of the seven surface lysine residues (Lys6, 11, 27, 29, 33, 48 or 63) on the previous Ub (Komander and Rape, 2012). Different multi‐Ub chains display different chemical and topological properties. For instance, the Lys48(K48)‐linked chain causes degradation of target proteins (Hershko and Ciechanover, 1998; Komander and Rape, 2012; Wickliffe *et al*., 2011), while the Lys63(K63)‐linked chain is usually involved in changing the activity of the target protein (Chen and Sun, 2009). Formation of the K63‐linked poly‐Ub chain requires a unique heterodimeric complex formed by the canonical E2, Ubc13, and another Ubc‐like protein named Ubc‐E2 variant (UEV) (Hofmann and Pickart, 1999; Romero‐Barrios and Vert, 2018).

In mammals, Ubc13‐mediated polyubiquitination of K63 plays an important role in the regulation of innate and adaptive immune responses, such as signal transduction and activating nuclear factor kappa enhancer‐binding protein (NF‐κB), which is a key immune modulator (Jiang and Chen, 2011; Wu and Karin, 2015). After signal recognition, a complex formed by Ubc13, Uev1A, and an E3 ubiquitin ligase (TRAF6) is responsible for catalysing the K63‐linked polyubiquitination of two kinase complexes, including TGFβ‐activated kinase 1 (TAK1) and IκB kinase (IKK). This modification promotes the phosphorylation of the IKK β subunit by TAK1, which in turn activates IKK. Knockout of *Ubc13* in experimental mice has been reported to result in autoimmunity (Chang *et al*., 2012), embryonic lethality (Fukushima *et al*., 2007; Yamamoto *et al*., 2006a) and defects in cell proliferation (Yamamoto *et al*., 2006b).

Different researchers have identified and cloned *Ubc13* and *UEV* family genes from several different plant species (Guo *et al*., 2015, 2016; Liu *et al*., 2021; Mural *et al*., 2013; Wang *et al*., 2017a; Wen *et al*., 2006, 2008; Zang *et al*., 2012), indicating that their functions should be conserved. In *Arabidopsis*, two Ubc13s (AtUbc13A/B) interact with all four UEVs (designated AtUEV1A‐D) and promote K63‐linked polyubiquitination *in vitro* (Wen *et al*., 2008). And these two *Ubc13* genes have been reported to be involved in the regulation of DNA damage repair (Wen *et al*., 2006), apical dominance (Yin *et al*., 2007), iron metabolism (Li and Schmidt, 2010) and auxin signalling (Wen *et al*., 2014). Recently, Wang *et al*. (2019) revealed that *Arabidopsis* Ubc13 differentially regulates two PCD pathways in responses to pathogen and low‐temperature stress. The *ubc13* double mutant exhibited hypothermia‐induced and salicylic acid (SA)‐dependent lesion‐mimicking phenotypes. But unlike typical lesion mimic mutants, *ubc13* did not show enhanced resistance to virulent bacterial and fungal pathogens (Wang *et al*., 2019). In addition, the tomato Ubc13 (Fni3/Sl‐Ubc13‐2) and UEV (Suv) have been found to positively regulate plant immunity through interaction with Fen, one protein kinase playing a pivotal role in ETI. The Ub‐conjugating activity of tomato Ubc13 and its interaction with Fen appear to be required for cell death triggered by Fen overexpression (OE) in *Nicotiana benthamiana* and by several NLR/effector pairs (Mural *et al*., 2013). In the rice genome, only one hypothetical gene (Os01g0673600) named *OsUbc13* showed high sequence identity with a pair of highly conserved *Arabidopsis Ubc13*. OsUbc13 has been identified to form stable heterodimers with all highly conserved UEVs (OsUEV1A–D) for mediating Ub chain assembly linked by K63 and also be required for tolerance to DNA damage (Wang *et al*., 2017a; Zang *et al*., 2012). Human ovarian tumour domain‐containing Ub aldehyde‐binding protein 1 (OTUB1) is a deubiquitinating enzyme, which can directly interact with Ubc13 and strongly suppress Ubc13‐dependent K63‐linked ubiquitination by preventing Ub transfer (Nakada *et al*., 2010; Wiener *et al*., 2012). Similarly, the interactions were detected *in vitro* and *in vivo* between the rice OsOTUB1.1 and OsUbc13 proteins (Wang *et al*., 2017b). However, whether OsUbc13 functions in rice innate immunity and disease resistance is unclear.

The sucrose non‐fermenting‐1 (SNF1) protein kinase family comprises SNF1 in *Saccharomyces cerevisiae*, the AMP‐activated protein kinase (AMPK) in mammals, and the SNF1‐related protein kinases (SnRKs) in higher plants. The conserved SNF1 family serves as the cellular energy sensor and regulator (Halford and Hey, 2009; Hardie, 2007; Hardie *et al*., 1998; Wang *et al*., 2021). In plants, SnRKs emerge as promising candidates to balance growth and defence (Cho *et al*., 2012; Kim *et al*., 2015; Nukarinen *et al*., 2016; Seo *et al*., 2011). These kinases form heterotrimeric complexes with a catalytic α‐subunit and regulatory β‐ and γ‐subunits. The α‐subunit consists of a highly conserved N‐terminal Ser/Thr kinase domain and a large C‐terminal regulatory domain that mediates its interaction with the β‐ and γ‐subunits (Broeckx *et al*., 2016; Hardie *et al*., 2012; Hedbacker and Carlson, 2008). The SnRK family of plants is subdivided into three subfamilies, SnRK1, SnRK2, and SnRK3, of which SnRK1 is most similar to SNF1 and AMPK (Coello *et al*., 2011; Ding *et al*., 2009; Hrabak *et al*., 2003). Increasing evidence has shown that SnRK1s play an important role in plants' immune responses. Plants overexpressing *SnRK1* are more resistant to geminivirus infection, while *SnRK1*‐silenced plants are more susceptible (Hao *et al*., 2003; Shen *et al*., 2011; Shen and Hanley‐Bowdoin, 2021). OE of *TaSnRK1α* in wheat plants increases the resistance against *F. graminearum* and silencing of *TaSnRK1α* has the opposite effect (Jiang *et al*., 2020). SnRK1 phosphorylates and destabilizes WRKY3 to enhance barley immunity to powdery mildew (Han *et al*., 2020). Rice has three genes encoding the α‐subunit of the SnRK1 complex, which can be subdivided into two subgroups, *OsSnRK1a* (*OSK1*) and *OsSnRK1b* (*OSK24*/*35*) (Takano *et al*., 1998). Among them, OSK35 positively regulates defence against *Magnaporthe oryzae* and *Xanthomonas oryzae* pv. *oryzae* (*Xoo*) (Kim *et al*., 2015). OsSnRK1a functions in the sugar signalling cascade (Lu *et al*., 2007) and energy homeostasis (Wang *et al*., 2021). Furthermore, OsSnRK1a has been reported to confer broad‐spectrum disease resistance and act as a master switch that regulates growth‐immunity trade‐offs in rice (Filipe *et al*., 2018), but its protein turnover and the mechanism by which OsSnRK1a‐mediated immunity is regulated remains largely unknown.

In this research, we investigated the molecular function of OsUbc13 in rice immunity. The *OsUbc13*‐RNA interference (RNAi) lines displayed lesion mimic phenotypes, accompanied by a significant increase in the accumulation of flg22‐ and chitin‐induced ROS, and in defence‐related gene expression or hormones. Unexpectedly, in contrast to the susceptibility caused by the *Arabidopsis Ubc13* mutation, knockdown of *OsUbc13* significantly increased rice resistance to *M*. *oryzae* and *Xoo*. Notably, OsUbc13 directly interacts with OsSnRK1a, which is the α catalytic subunit of SnRK1 (sucrose nonfermentation 1‐related protein kinase 1) and positively regulates broad‐spectrum disease resistance in rice. In the *OsUbc13*‐RNAi plants, although the protein abundance of OsSnRK1a did not change, its activity and sensitivity to ABA were obviously enhanced, and K63‐linked polyubiquitination was weaker than that of wild‐type Dongjin (DJ). OE of the deubiquitinase‐encoding gene *OsOTUB1.1* produced similar effects to the inhibition of *OsUbc13* in affecting immunity responses, *M*. *oryzae* resistance, OsSnRK1a ubiquitination, and OsSnRK1a activity. Furthermore, re‐interfering with *OsSnRK1a* in one *OsUbc13*‐RNAi line (Ri‐3) partially restored its *M*. *oryzae* resistance to a level between those of Ri‐3 and DJ. Our data demonstrate OsUbc13 negatively regulates immunity against pathogens by enhancing the activity of OsSnRK1a.

## Results

### Phenotypic characterization of the *OsUbc13*‐RNAi lines

**Figure 1:**
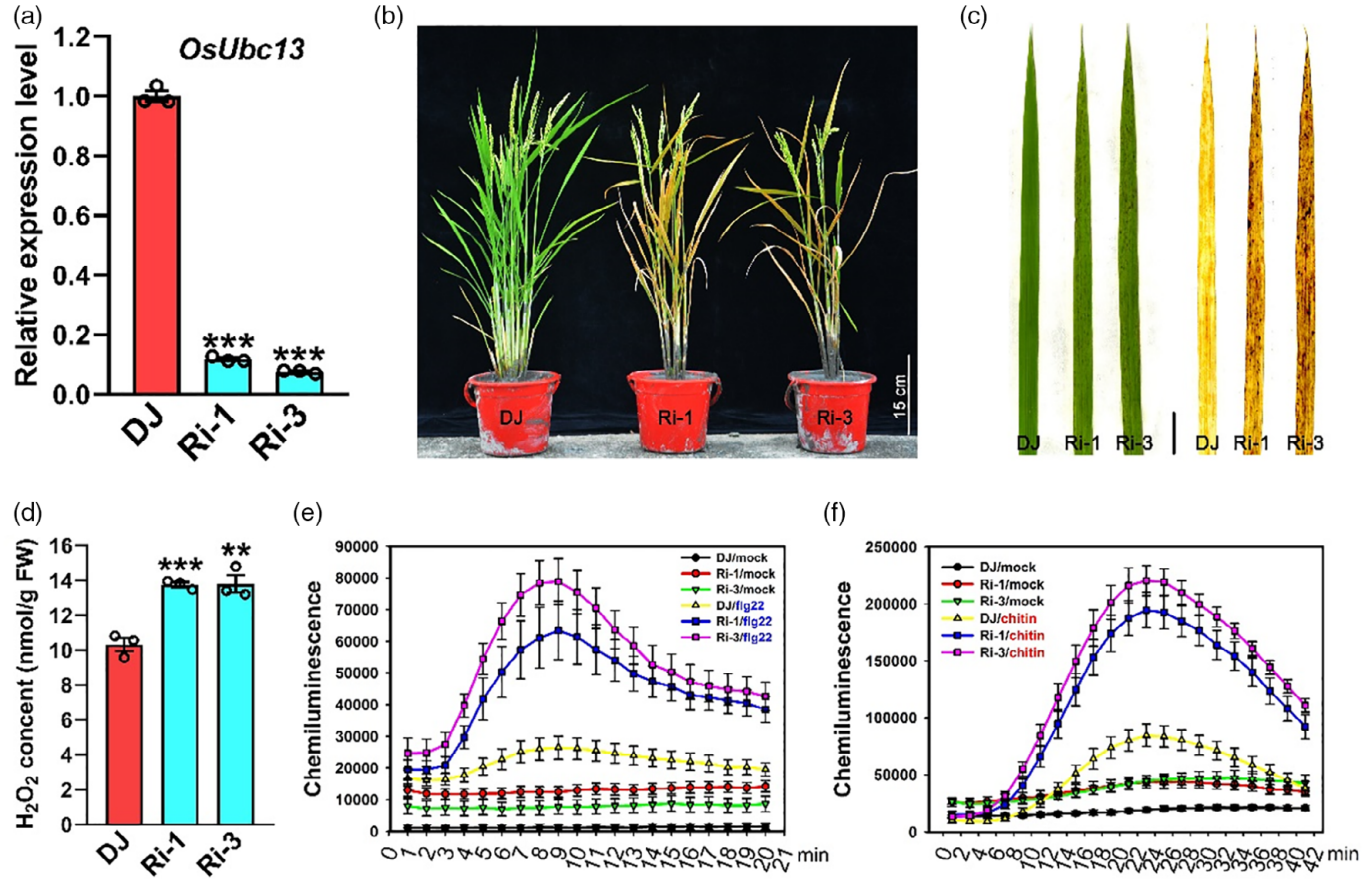
The *OsUbc13*‐RNAi lines exhibit lesion mimic phenotype accompanied with reactive oxygen species burst and accelerated leaf senescence. (a) qRT‐PCR analysis of *OsUbc13* expression in two *OsUbc13*‐RNAi (Ri‐1 and Ri‐3) lines. *OsActin1* gene was used as an internal control. Data are shown as means ±SE; *n* = 3 (***P < 0.001; Student's *t*‐test). (b) Whole plants of the wild‐type DJ and two *OsUbc13*‐RNAi lines at the heading and grain filling stage in the paddy field. Scale bar = 15 cm. (c) Lesion mimic phenotypes and DAB staining on leaves of the *OsUbc13*‐RNAi plants at 30‐day post‐sowing in soil, compared with that of DJ. Scale bar = 1 cm. (d) Detection of the H_2_O_2_ content of DJ and the *OsUbc13*‐RNAi plants at 30‐day post‐sowing in soil. Data are shown as means ±SE; *n* = 3 (**P < 0.01, ***P < 0.001; Student's *t*‐test). (e) ROS accumulation dynamics in DJ and *OsUbc13*‐RNAi plants after flg22 and water (mock) treatments. Data are shown as means ±SE; *n* = 3. (f) ROS accumulation dynamics in DJ and *OsUbc13*‐RNAi plants after chitin and water (mock) treatments. Data are shown as means ±SE; *n* = 3.

To survey the potential function of OsUbc13 in rice innate immunity and disease resistance, 15 independent RNA interference (RNAi) lines of *OsUbc13* were successfully produced. Two lines (Ri‐1 and Ri‐3), in which the relative expression level of *OsUbc13* was approximately one‐tenth of that in wild‐type DongJin (DJ, Figure 1a), were selected for follow‐up studies. Under field conditions in summer (Fuzhou, China), the tips of lower (older) leaves of the two *OsUbc13*‐RNAi lines exhibited irregular brown necrotic lesions approximately 30 days post‐sowing. The lesions then spread gradually, eventually extending to whole leaves, leading to severe leaf withering and premature senescence (Figure 1b,c). Cell death lesions eventually appeared on the top of *OsUbc13*‐RNAi plants, and by the heading and grain‐filling stage, these gene‐silenced plants exhibited a pronounced senescence phenotype (Figure 1b). When *OsUbc13*‐RNAi lines were grown in the greenhouse, less severe lesions were visible about 40 days after planting in soil, and longer in the nutrient solution. These variations in both the timing of lesion appearance and in lesion severity suggest that cell death in *OsUbc13*‐RNAi plants is influenced by growth conditions. In addition to cell death and premature senescence, two key agronomic traits including tiller number and grain number per panicle were adversely affected in *OsUbc13*‐RNAi plants (Figure S1), which is consistent with perturbation of the *OsUbc13* gene in the Zhonghua11 (ZH11) background by another research group (Wang *et al*., 2017b). To determine whether the development of lesions in the *OsUbc13*‐RNAi lines involves altered hydrogen peroxide (H_2_O_2_) accumulation, we performed 3,3′‐diaminobenzidine (DAB) staining. As shown in Figure 1c, strong brown staining is prominent around leaf lesions in *OsUbc13*‐RNAi plants, as opposed to in the leaves of wild‐type DJ, indicating that a significant amount of H_2_O_2_ was accumulated when *OsUbc13* was silenced. We further quantified that the concentration of H_2_O_2_ in *OsUbc13*‐RNAi is significantly higher than that in wild‐type DJ plants (Figure 1d), and so is the production of malondialdehyde (MDA, Figure S2a), another biomarker related to cell death and lipid peroxidation (Ayala *et al*., 2014; Ruan *et al*., 2019). Photosynthetic pigment content was measured to check the effect of lesions on *OsUbc13*‐RNAi plants. The levels of chlorophyll a, chlorophyll b and carotenoids were significantly reduced in Ri‐1 and Ri‐3 compared with DJ (Figure S2b), suggesting that the formation of necrotic lesions had a negative effect on photosynthetic pigment content, which might be the cause of poor agronomic performance of the *OsUbc13*‐RNAi lines.

To further investigate whether the ROS signalling pathway is impaired in *OsUbc13*‐RNAi plants, we compared the dynamics of flg22‐ or chitin‐induced ROS generation using a chemical luminescence assay. The ROS production rate was faster in *OsUbc13*‐RNAi than in DJ in response to both flg22 and chitin treatments (Figure 1e,f). ROS levels peaked ~9 min after flg22 treatment, and these levels were nearly 2.40 or 2.99 times higher in Ri‐1 or Ri‐3 than in DJ, respectively (Figure 1e). With chitin treatment, the ROS levels peaked ~23 min, and the levels were nearly 2.30 or 2.60 times higher in Ri‐1 or Ri‐3 than in DJ, respectively (Figure 1f). Even in the control treated with water, the basal ROS level was also higher in Ri‐1 and Ri‐3 than in DJ (Figure 1e,f). Taken together, these results indicate that the ROS signalling pathway is enhanced in *OsUbc13*‐RNAi plants.

### Enhanced resistance of *OsUbc13*‐RNAi plants against *Magnaporthe oryzae* and *Xanthomonas oryzae pv. oryzae*

**Figure 2:**
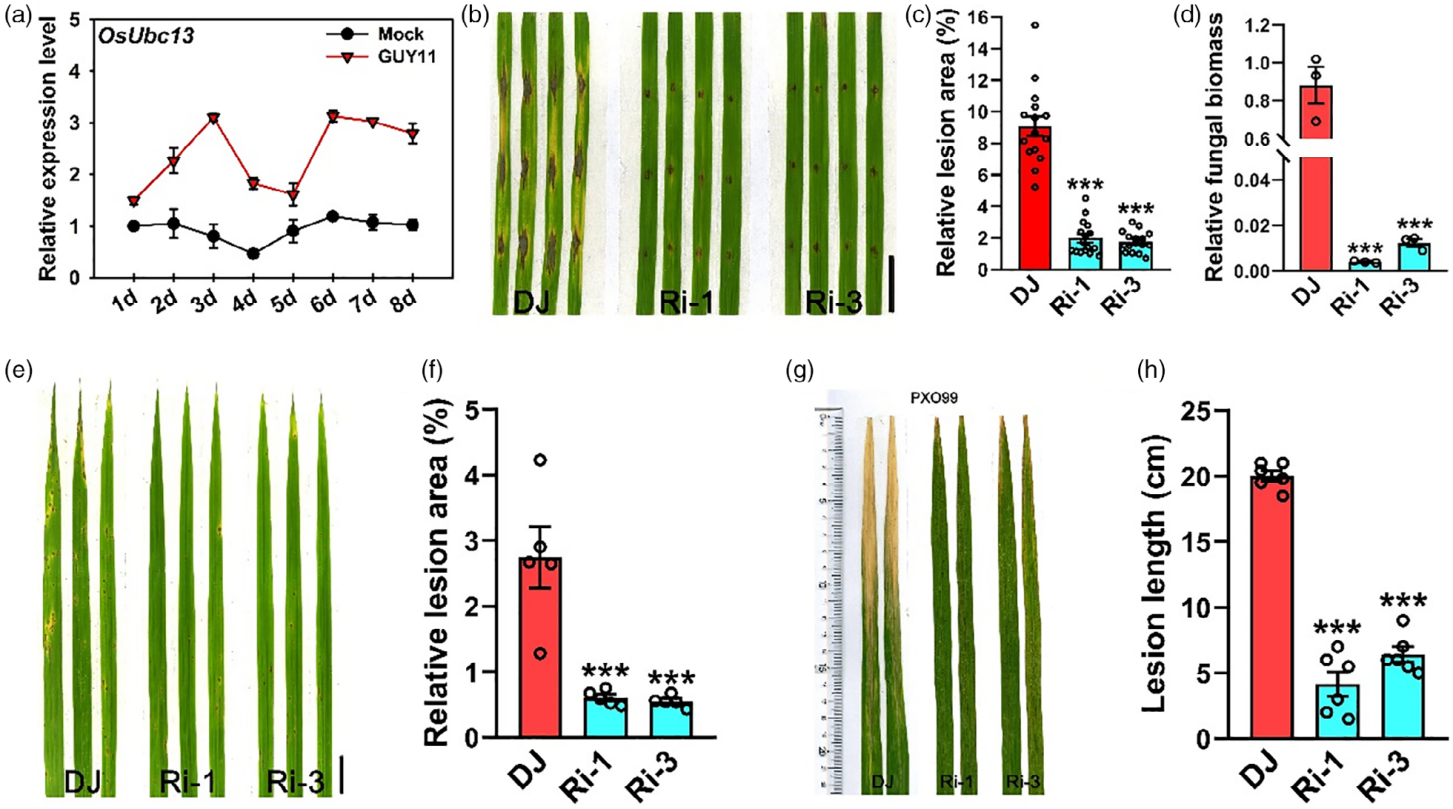
The *OsUbc13*‐RNAi lines display enhanced resistance to both *M. oryzae* and *Xoo*. (a) qRT‐PCR analysis of *OsUbc13* expression level at different time after inoculation with the compatible isolate GUY11 of *M. oryzae*. Two‐week‐old wild‐type DJ seedlings were used for inoculation. The seedlings sprayed only with 0.025% Tween 20 were used as negative control (Mock). *OsActin1* gene was used as an internal control. Data are shown as means ±SE; *n* = 3. (b) The lesions on DJ and *OsUbc13*‐RNAi leaves at 8 days after punch inoculation with the compatible *M. oryzae* isolate GUY11. Scale bar = 1 cm. (c) Relative lesion area (%) in leaves of (b) indicates significant differences between DJ and *OsUbc13*‐RNAi. Data are shown as means ±SE; *n* = 15 (***P < 0.001; Student's *t*‐test). (d) Relative fungal biomass, measured as *MoPot2* by qRT‐PCR, in leaves of (b) was normalized to *OsUbq* DNA (Park *et al*., 2012). Data are shown as means ±SE; *n* = 3 (***P < 0.001; Student's *t*‐test). (e) The lesions on DJ and *OsUbc13*‐RNAi leaves at 8 days after spraying inoculation with the compatible *M. oryzae* isolate GUY11. Scale bar = 1 cm. (f) Relative lesion area (%) in leaves of (e) indicates significant differences between DJ and *OsUbc13*‐RNAi. Data are shown as means ±SE; *n* = 5 (***P < 0.001; Student's *t*‐test). (g) The lesions on DJ and *OsUbc13*‐RNAi leaves at 18 days after inoculation with the compatible *Xoo* isolate PXO99. (h) Lesion lengths in leaves of (g) indicate significant differences between DJ and *OsUbc13*‐RNAi. Data are shown as means ±SE; *n* = 6 (***P < 0.001; Student's *t*‐test).

Given that *OsUbc13*‐RNAi had hypersensitivity‐like lesions and more ROS accumulation after treatment with PAMPs (flg22 and chitin), we speculated that these transgenic plants may exhibit enhanced resistance to rice pathogens. Before testing this hypothesis, we first examined the response of the *OsUbc13* gene to *M. oryzae* infection through quantitative reverse transcription PCR (qRT‐PCR). As illustrated in Figure 2a, the expression level of *OsUbc13* in wild‐type DJ seedlings was induced by inoculation with GUY11, a virulent isolate of *M*. *oryzae*; the induced expression reached a peak at 3‐ and 6‐day post‐inoculation, indicating that OsUbc13 may participate in rice responses to *M*. *oryzae* infection. To evaluate the blast resistance, detached leaves of 1‐month‐old seedlings of DJ and *OsUbc13*‐RNAi were inoculated with GUY11 using the punch method in a controlled environment. Although the *OsUbc13*‐RNAi plants growing in greenhouse did not display lesion mimic phenotypes when challenged with GUY11, both relative lesion area and fungal biomass in the lesions of which were significantly lower than in DJ (Figure 2bd). The disease resistance test was repeated with a spraying inoculation method using conidia. The two *OsUbc13*‐RNAi lines develop only small, scattered lesions compared with DJ which shows typical blast lesions (Figure 2e,f). In another test, fully expanded flag leaves of DJ and *OsUbc13*‐RNAi were inoculated with one bacterial blight pathogen *Xoo* strain PXO99. At 15‐day post‐inoculation, the lesion lengths were much shorter on *OsUbc13*‐RNAi plants than on DJ plants (Figure 2g,h). These results demonstrate that the *OsUbc13*‐RNAi plants display significantly enhanced resistance to both *M. oryzae* and *Xoo*. In addition to RNAi lines, we also tried to generate knockout lines using the CRISPR/Cas9 method (Ma *et al*., 2015) to investigate OsUbc13's function in disease resistance. However, we were unable to obtain successful transgenic plants, probably due to the lethality of knocking out *OsUbc13*.

OE lines of the *OsUbc13* gene were successfully constructed (Figure S3a). In contrast to the RNAi lines, the two OE lines (OE17‐2 and OE18‐3) did not exhibit obvious reduced or enhanced resistance to *M. oryzae* compared with DJ (Figure S3b,c). These results demonstrate that the disruption of *OsUbc13* is responsible for enhanced disease resistance.

**Figure 3:**
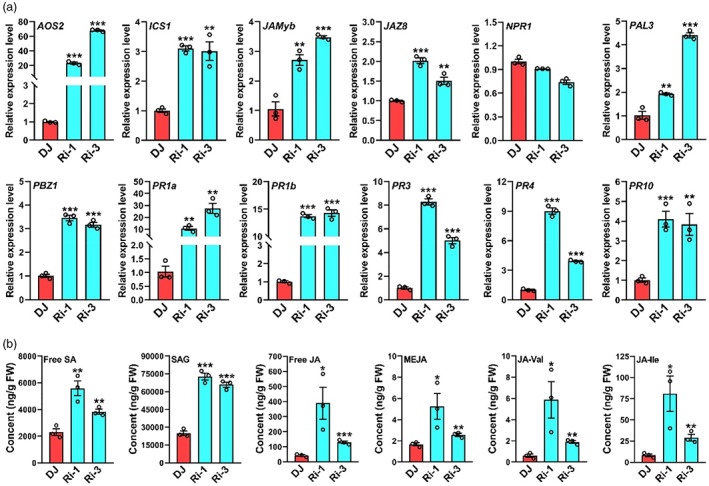
Constitutive expression of several defence‐related genes and contents of defence phytohormones in *OsUbc13*‐RNAi and DJ. (a) qRT‐PCR was used to analyse the expression of defence‐related genes involved in JA or SA signalling/synthetic pathway. Total RNA was extracted from the leaves of *OsUbc13*‐RNAi and DJ plants at 30‐day post‐sowing in soil. *OsActin1* gene was used as an internal control. Data are shown as means ±SE; *n* = 3 (**P < 0.01, ***P < 0.001; Student's *t*‐test). (b) Contents of free salicylic acid (SA), salicylic acid 2‐O‐β‐glucoside (SAG), free jasmonic acid (JA), methyl jasmonate (MEJA), N‐[(−)‐Jasmonoyl]‐(L)‐valine (JA‐Val), and jasmonoyl‐L‐isoleucine (JA‐Ile) in *OsUbc13*‐RNAi and DJ. The leaves of *OsUbc13*‐RNAi and DJ plants at 30‐day post‐sowing in soil were used to extract SA, SAG, JA, MEJA, JA‐Val, and JA‐Ile. Data are shown as means ±SE; *n* = 3 (*P < 0.05, **P < 0.01, ***P < 0.001; Student's *t*‐test).

To ascertain whether enhanced disease resistance response stemmed from elevated expression of defence‐related genes, we examined their expression by qRT‐PCR. These defence genes mainly consisted of those involved in jasmonic acid (JA) and SA signalling and/or biosynthesis. As components of the JA signalling, *JAMyb*, *PBZ1*, and *JAZ8* genes (Sathe *et al*., 2019) were highly expressed in Ri‐1 and Ri‐3 compared with that in DJ. *PAL3*, *PR1a*, *PR1b*, *PR3*, *PR4*, and *PR10*, which are known to be involved in the SA signalling pathway (Park *et al*., 2012), were all also significantly up‐regulated in Ri‐1 and Ri‐3 (Figure 3a). Notably, in Ri‐1 and Ri‐3, the expression of *AOS2*, which encodes allene oxide synthase (a key enzyme in the JA biosynthetic pathway), was increased to about 25‐ and 70‐fold higher than that in DJ, respectively; the expression of *ICS1*, which encodes isochorismate synthase (a key enzyme in the SA biosynthetic pathway), was elevated to about threefold (Figure 3a). Furthermore, the abundance of various forms of SA and JA, including free SA, SA 2‐O‐β‐Glucoside (SAG), free JA, methyl jasmonate (MEJA), N‐[(−)‐jasmonoyl]‐(L)‐valine (JA‐Val), and N‐[(−)‐jasmonoyl]‐(S)‐isoleucine (JA‐Ile, the active form of JA) was stronger in *OsUbc13*‐RNAi than that in DJ (Figure 3b). Collectively, these data suggest that *OsUbc13*‐RNAi lines confer enhanced disease resistance by enhancing flg22‐ and chitin‐triggered ROS accumulation and possibly by activating the SA and JA signalling pathways in rice.

### OsUbc13 interacts with OsSnRK1a

The identification and in‐depth study of OsUbc13‐interacting proteins are crucial to revealing its molecular mechanisms regulating innate immunity and disease resistance in rice. Coincidentally, in our previous study, 20 potential OsUbc13‐interacting partners have been found through yeast two‐hybrid (Y2H) assay (Wang *et al*., 2014). Based on predicted protein function and existing literature reports, among these candidate proteins, Os10g0445500 (hypersensitive response‐like lesion‐inducer family protein, named by us as OsHRLI), Os05g0409300 (similar to cysteine protease inhibitor, named by us as OsCPI) and Os08g0199300 (similar to YyaF/YCHF TRANSFAC/OBG family small GTpase plus RNA binding domain TGS, known and named as OsYchF1) may be related to the disease resistance of rice (Cheung *et al*., 2016; Wang *et al*., 2014; Zhang *et al*., 2020a). Os05g053050 (*Oryza sativa* S‐phase kinase‐associated protein 1‐like protein 1, known and named as OsSnRK1a) has been confirmed to have the function of improving broad‐spectrum disease resistance in rice (Filipe *et al*., 2018; Wang *et al*., 2014).

**Figure 4:**
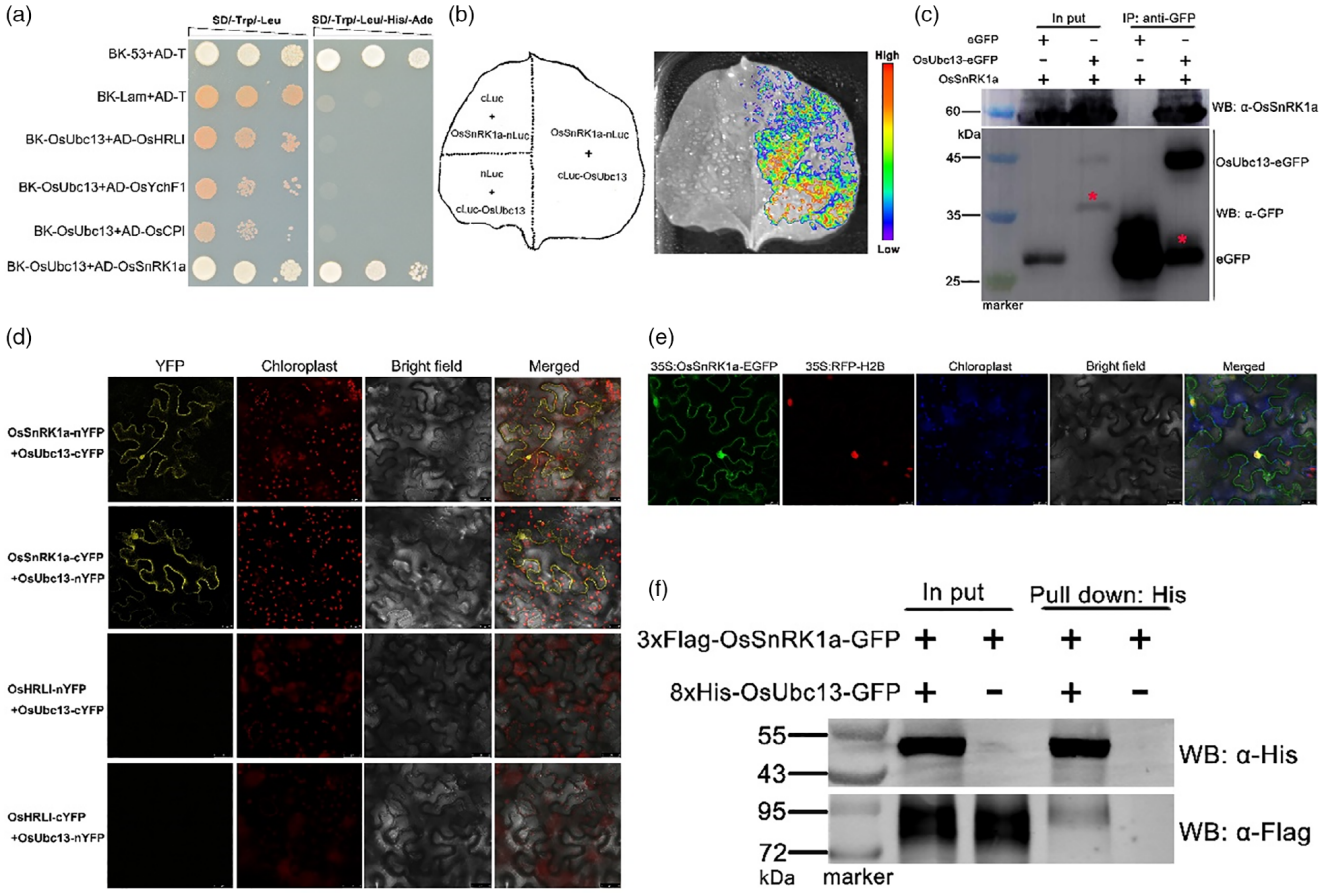
OsUbc13 interacts with OsSnRK1a. (a) Y2H assay. The pGBKT7 plasmid containing the OsUbc13 coding sequence (BK‐OsUbc13) and the pGADT7 plasmid containing the OsHRLI, OsYchF1, OsCPI, and OsSnRK1a coding sequence (AD‐OsHRLI, ‐OsYchF1, ‐OsCPI, ‐OsSnRK1a) were co‐transformed into yeast cells (AH109). Yeast cells co‐transformed with AD‐T/BK‐53 or AD‐T/BK‐Lam vectors were used as the positive or negative control, respectively. Interaction of OsUbc13 with OsSnRK1a was indicated by the ability of yeast cells to grow on dropout medium lacking Leu, Trp, His, and Ade for 5 days after plating. (b) LCI assay. Agrobacterial strains containing different combinations of plasmids were co‐infiltrated into tobacco leaves. A cooled charge‐coupled imaging apparatus was used to capture the images. No signal was obtained for the negative controls in which OsSnRK1a‐nLuc was co‐expressed with cLuc, and cLuc‐OsUbc13 was co‐expressed with nLuc. The pseudocolor bar indicates the range of luminescence intensity. (c) Co‐IP assay. OsUbc‐eGFP and OsSnRK1a were co‐expressed in tobacco leaves to detect the interaction between OsUbc13 and OsSnRK1a. An anti‐GFP affinity matrix was used for immunoprecipitation and anti‐OsSnRK1a was used for immunoblot analysis. Red asterisks indicate nonspecific bands. Experiments were repeated two times with similar results. (d) BiFC assay. Fluorescence was observed in the nuclear compartment of transformed tobacco (*N. benthamiana*) cells, resulting from the complementation of OsSnRK1a‐nYFP+OsUbc13‐cYFP or OsSnRK1a‐cYFP+OsUbc13‐nYFP. No signal was obtained for the negative controls in which OsHRLI‐nYFP was co‐expressed with OsUbc13‐cYFP, and OsHRLI‐cYFP was co‐expressed with OsUbc13‐nYFP. YFP signal was detected by confocal microscopy. Scale bars = 25 μM. (e) Subcellular localization of OsSnRK1a in leaves of tobacco (*N. benthamiana*). EGFP, enhanced green fluorescent protein; RFP‐H2B, a nuclear marker. Scale bars = 25 μM. (f) Pull‐down assay. 3 × Flag‐OsSnRK1a‐GFP and 8 × His‐OsUbc13‐GFP recombinant proteins expressed by CFPS (cell‐free protein synthesis) reactions were used in the pull‐down assay. Equal amounts of 3 × Flag‐OsSnRK1a‐GFP and 8 × His‐OsUbc13‐GFP were incubated with His‐tag magnetic beads. Pull‐down products were detected using anti‐His and anti‐Flag antibodies. Experiments were repeated two times with similar results.

To further confirm the interactions of these four proteins with OsUbc13, we performed point‐to‐point Y2H analysis. The full‐length coding sequence (CDS) of *OsUbc13* gene was ligated with the Gal4 DNA‐binding domain of the bait vector pGBKT7 (BK‐OsUbc13); the full‐length CDS of *OsHRLI*, *OsCPI*, *OsYchF1*, or *OsSnRK1a* gene was fused to the Gal4 activation domain (AD) of the prey vector pGADT7 (AD‐OsHRLI, AD‐OsCPI, AD‐ OsYchF1, and AD‐OsSnRK1a). As shown in Figure 4a, OsUbc13 is only physically associated with OsSnRK1a in the Y2H system, but not with the other three proteins. Next, a firefly split‐luciferase complementation imaging (LCI) assay was carried out to consolidate the evidence for the interaction between the two. The N‐terminal of firefly luciferase (nLuc) was fused with OsSnRK1a, whereas the C‐terminal (cLuc) was connected to OsUbc13. OsSnRK1a‐nLuc and cLuc‐OsUbc13 were transiently co‐expressed in wild‐type tobacco (*N. benthamiana*) leaves. OsSnRK1a‐nLuc/cLuc and cLuc‐OsUbc13/nLuc were used as negative controls. A strong fluorescence signal was observed in leaves co‐expressing OsSnRK1a‐nLuc and cLuc‐OsUbc13, but not in the leaves expressing plasmids for negative controls (Figure 4b). Furthermore, co‐immunoprecipitation (Co‐IP) assays in tobacco leaves also confirmed that OsUbc13 interacts with OsSnRK1a *in vivo*, because we found that OsSnRK1a co‐precipitated with OsUbc13‐eGFP but not with the control eGFP (Figure 4c). Bimolecular fluorescence complementation (BiFC) experiments were performed to determine where OsUbc13 and OsSnRK1a interact in plant cells. The N‐ and C‐terminal of yellow fluorescent protein (YFP) were coupled to OsSnRK1a or OsUbc13 (OsSnRK1a‐nYFP/cYFP and OsUbc13‐nYFP/cYFP), respectively. And then, these recombinant vectors were transiently expressed in leaf cells of wild‐type tobacco (*N. benthamiana*). The strong YFP signal was visualized mainly from the OsSnRK1a‐nYFP/OsUbc13‐cYFP and OsSnRK1a‐cYFP/OsUbc13‐nYFP complementation assay in the cytoplasm and nucleus of the transformed cells, but not in the negative controls (Figure 4d). At the same time, we found that OsSnRK1a was mainly localized to the cytoplasm and nucleus (Figure 4e), which is consistent with previous findings and OsUbc13's localization (Cho *et al*., 2012; Liu *et al*., 2021; Wang *et al*., 2014, 2021). Interaction of OsSnRK1a and OsUbc13 in the yeast or plant cells could be either direct or indirect, possibly involving an E3 ligase. To distinguish between these possibilities, we used a pull‐down assay to examine the binding of OsUbc13 to OsSnRK1a *in vitro*, in which the 3 × Flag‐OsSnRK1a‐GFP and 8 × His‐OsUbc13‐GFP recombinant proteins were obtained from a cell‐free protein synthesis system (Zhang *et al*., 2020b). The results showed that the interaction between OsUbc13 and OsSnRK1a does not require other proteins and is direct (Figure 4f). Taken overall, we conclude that OsUbc13 directly interacts with OsSnRK1a both in vivo and in vitro.

### The C‐terminal fragment of OsSnRK1a and the 89th cysteine residue of OsUbc13 are responsible for their interaction

**Figure 5:**
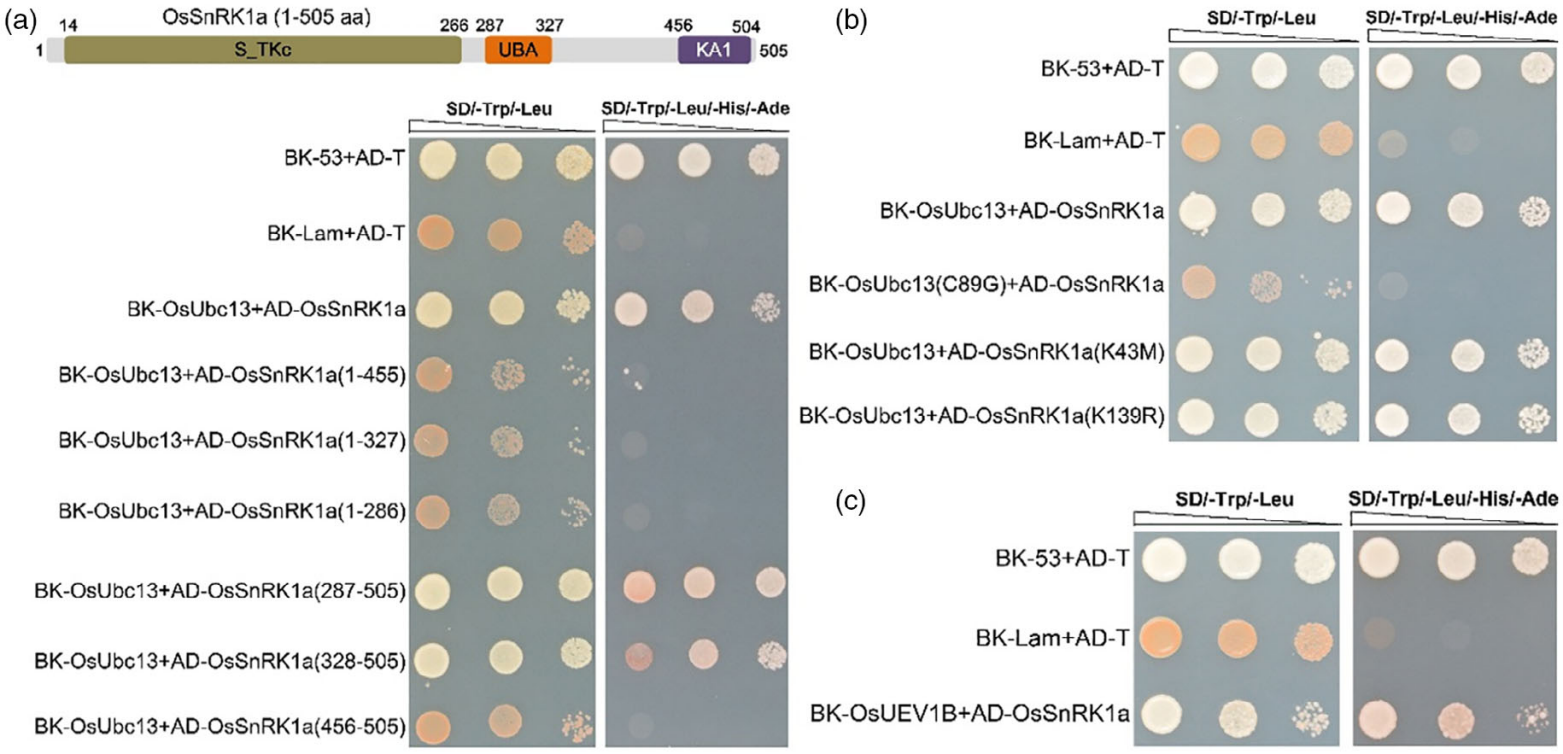
Y2H screening assay to identify necessary sites and regions required for the interaction between OsUbc13 and OsSnRK1a. (a) Interaction test between OsUbc13 and different truncated OsSnRK1a proteins with specific deletion. Top, the schematic diagram of OsSnRK1a protein. S_TKc, serine/treonine kinase catalytic domain; UBA, ubiquitin associated domain (UBA); KA1, kinase‐associated 1 domain. Low, the interaction was indicated by the ability of yeast cells to grow on dropout medium lacking Leu, Trp, His, and Ade for 5 days after plating. Yeast cells co‐transformed with AD‐T/BK‐53 or AD‐T/BK‐Lam vectors were used as the positive or negative control, respectively. (b) Interaction test between OsUbc13(C89G) and OsSnRK1a, or between OsUbc13 and OsSnRK1a(K43M)/(K139R). Interaction was indicated by the ability of yeast cells to grow on dropout medium lacking Leu, Trp, His, and Ade for 5 days after plating. Yeast cells co‐transformed with AD‐T/BK‐53 or AD‐T/BK‐Lam vectors were used as the positive or negative control, respectively. (c) OsUEV1B interacts with OsSnRK1a. Interaction was indicated by the ability of yeast cells to grow on dropout medium lacking Leu, Trp, His, and Ade for 5 days after plating. Yeast cells co‐transformed with AD‐T/BK‐53 or AD‐T/BK‐Lam vectors were used as the positive or negative control, respectively.

As reported previously (Emanuelle *et al*., 2016; Filipe *et al*., 2018), the OsSnRK1a protein, consisting of 505 amino acid residues, comprises an amino (N)‐terminal serine/treonine kinase catalytic domain (S_TKc) followed by a ubiquitin‐associated domain (UBA) and a kinase‐associated 1 (KA1) domain at its carboxyl (C)‐terminal (Figure 5a, top). To identify the region of OsSnRK1a essential for the interaction with OsUbc13, we fused six truncated OsSnRK1a variants to the Gal4 AD of the prey vector and examined the interactions of these variants with OsUbc13 by Y2H analysis. As shown in Figure 5a, deletion of the C‐terminal amino acid residues 456–505, 328–505, or 287–505 of OsSnRK1a (AD‐OsSnRK1a^1–455^, AD‐OsSnRK1a^1–327^, and AD‐OsSnRK1a^1–286^) completely abolished the OsUbc13‐OsSnRK1a interaction, whereas only retaining the 219 or 178 residues at the C‐terminus of OsSnRK1a that harbour the KA1 domain (AD‐OsSnRK1a^287–505^ and AD‐OsSnRK1a^328–505^) did not affect the OsUbc13‐OsSnRK1a interaction. This result showed that the C‐terminal region of OsSnRK1a is required for its interaction with OsUbc13. Further mapping revealed that 178 amino acids spanning the C‐terminal KA1 domain (OsSnRK1a^328–505^), rather than just the KA1 domain, are exclusively involved in the OsUbc13‐OsSnRK1a interaction because an OsSnRK1a variant in which the N‐terminal amino acids 1–455 were deleted and only the KA1 domain was retained (AD‐OsSnRK1a^456–505^) could not physically associate with OsUbc13 (Figure 5a).

Zang *et al*. (2012) demonstrated that OsUbc13 shows a high degree of conservation with Ubc13s from other eukaryotic organisms. As might be expected with this overall high level of amino acid sequence similarity between OsUbc13 and other Ubc13 homologues, it has all of the functionally important amino acids for biochemical activities of Ubc13, namely, Cys‐89 located in the putative active site for ubiquitin thioester formation, Met‐66, which is involved in the interaction with an E3 ligase, and three pocket residues (Glu‐57, Phe‐59, and Arg‐72) that determine binding specificity for Uev protein (VanDemark *et al*., 2001; Wooff *et al*., 2004). The positions of these amino acids are strictly identical to those in Fni3 (Figure S4), a homologue of the Ubc13‐type ubiquitin‐conjugating enzyme in tomato that catalyses exclusively K63‐linked ubiquitination (Mural *et al*., 2013). Substitution of Cys‐89 in the active site of Fni3 with Gly (Fni3^C89G^) essentially abolishes the Fen‐Fni3 interaction (Mural *et al*., 2013). As expected, the substitution of Cys‐89 of OsUbc13 (OsUbc13^C89G^) almost completely eliminated the interaction between OsUbc13 and OsSnRK1a in the Y2H system (Figure 5b). To validate the protein kinase (PK) function of OsSnRK1a in the OsSnRK1a‐OsUbc13 interaction, inactive forms of OsSnRK1a were prepared as ATP binding site‐mutated PK (OsSnRK1a^K43M^, substitution of Lys‐43 with Met) and as catalytically inactive PK (OsSnRK1a^K139R^, substitution of Lys‐139 with Arg) (Cho *et al*., 2012). As shown in Figure 5b, mutation of Lys‐43 or Lys‐139 in OsSnRK1a had no difference on the interaction between OsSnRK1a and OsUbc13 in the Y2H system. These results suggest that the OsUbc13‐OsSnRK1a interaction may mainly depend on the ubiquitin‐conjugating enzyme activity of OsUbc13 rather than the kinase activity of OsSnRK1a.

Formation of the K63‐linked poly‐Ub chain requires Ubc13 and another Ubc‐like protein named UEV (Hofmann and Pickart, 1999; Romero‐Barrios and Vert, 2018). In our recent study, OsUEV1B has been reported to interact with OsUbc13 and be required for phosphate homeostasis in rice (Liu *et al*., 2021). According to this, we speculated that OsUEV1B can also associate with OsSnRK1a to assist OsUb13 in the K63‐linked ubiquitination of OsSnRK1a. As shown in Figure 5c, the Y2H assay proved our hypothesis that OsUEV1B does interact with OsSnRK1a. However, unlike the *OsUbc13*‐RNAi lines, the *osuev1b* mutant did not show significantly improved resistance to rice blast (Figure S5), which may be related to the functional redundancy of the OsUEV1 protein family. As a potential target of OsUbc13‐OsUEV1B complex, OsVDAC1, a voltage‐gated anion channel protein, functions in maintaining phosphorus homeostasis in rice (Liu *et al*., 2021), but the *osvdac1* mutant also displayed similar blast sensitivity with wild‐type DJ (Figure S5). These findings drove us to focus our next studies on OsSnRK1a.

### Silencing *OsUbc13* attenuates K63‐linked polyubiquitination of OsSnRK1a and improves SnRK1 activity

**Figure 6:**
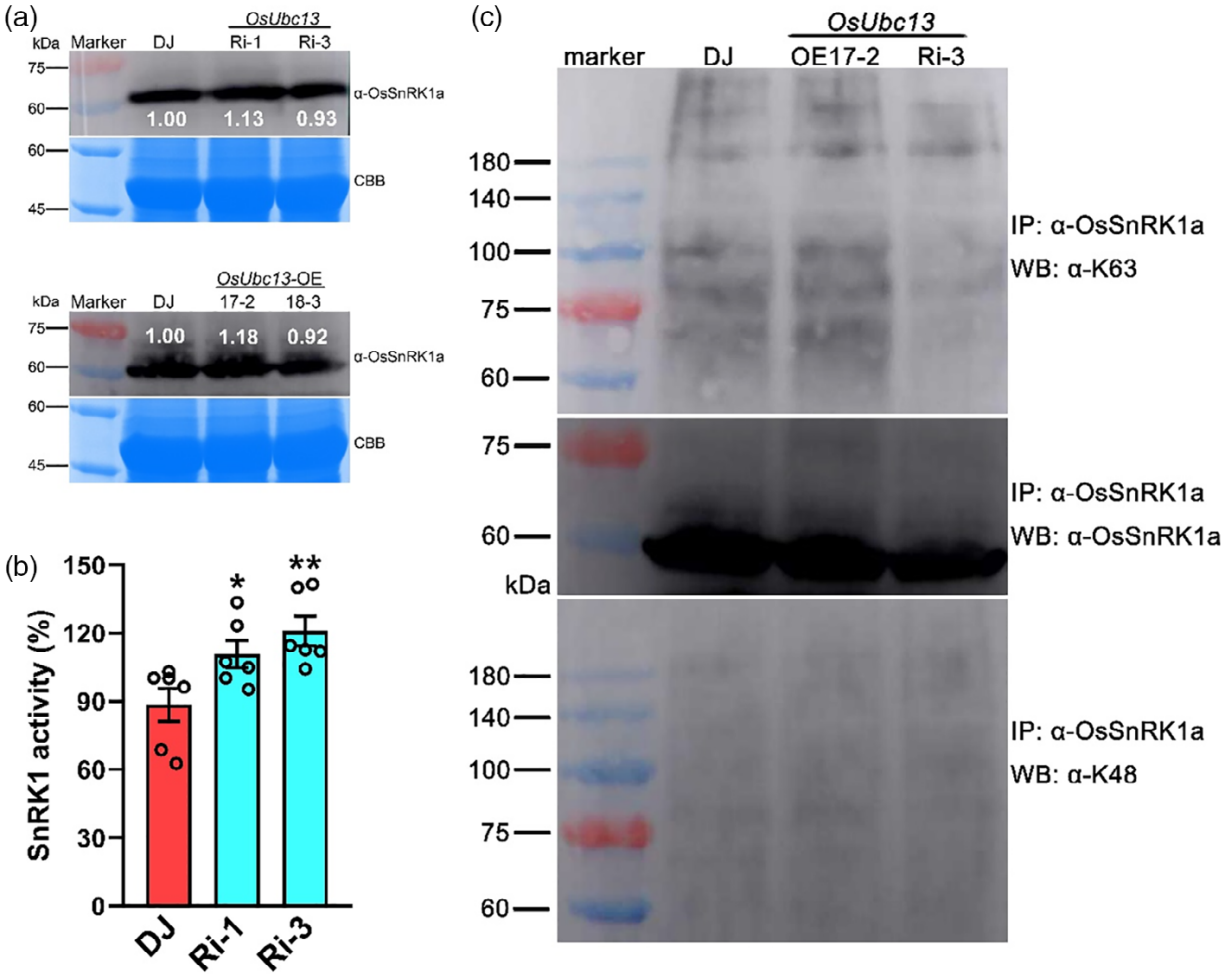
Detection of OsSnRK1a protein content, SnRK1 activity, and polyubiquitination degree. (a) The protein level of OsSnRK1a in leaves of DJ, *OsUbc13*‐RNAi and ‐OE plants at 30‐day post‐sowing in soil was detected using anti‐OsSnRK1a. Coomassie brilliant blue (CBB) staining was used as a loading control. Band intensity was calculated by ImageJ software. Experiments were repeated three times with similar results. (b) The SnRK1 kinase activity in leaves of DJ and *OsUbc13*‐RNAi plants at 30‐day post‐sowing in soil. Data are shown as means ±SE; *n* = 6 (*P < 0.05, **P < 0.01; Student's *t*‐test). (c) *In vivo* polyubiquitination level of OsSnRK1a in DJ, *OsUbc13*‐OE line (OE17‐2), and OsUbc13‐RNAi line (Ri‐3). Crude protein extracted from seedlings at 30‐day post‐sowing in soil was immunoprecipitated by anti‐OsSnRK1a antibody and detected using antibodies that specifically recognize K48/K63‐polyubiquitin conjugates (anti‐K48 and K63) and anti‐OsSnRK1a antibody. Experiments were repeated two times with similar results.

Although in a different background, silencing of *OsUbc13* by RNAi resulted in phenotypes similar to those of plants overexpressing *OsSnRK1a* (Filipe *et al*., 2018), at least with respect to the resistance to *M. oryzae* and *Xoo* and the number of tillers per plant (Figures 2b–h and S1b). Given the interaction between OsUbc13 and OsSnRK1a, we speculated that there were at least two possibilities for the emergence of these similar phenotypes. First, the protein level of OsSnRK1a was increased in the *OsUbc13*‐RNAi line; second, the activity of OsSnRK1a was enhanced. Both will help to increase the activity of the SnRK1 complex. Growing evidence suggests that OE of the α catalytic subunit of the SnRK1 complex can enhance total SnRK1 activity (Jossier *et al*., 2009; Wang *et al*., 2020; Yu *et al*., 2018). In our study, transient expression of the OsSnRK1a‐EGFP fusion protein in tobacco leaves increased the total SnRK1 activity by about 34.05% compared with the expression of empty EGFP alone (Figure S6). To test the first possibility, we detected the protein level of OsSnRK1a using anti‐OsSnRK1a antibody. OsSnRK1a protein level was similar to that of wild‐type DJ in both the interference and OE lines of *OsUbc13*, and there was no obvious change (Figure 6a). Therefore, the first possibility might be ruled out. Likewise, silencing or OE of *OsUbc13* had no significant effect on the transcription of *OsSnRK1a* (Figure S7).

To test another possibility, we directly measured SnRK1 activity in seedlings of *OsUbc13*‐RNAi lines. As shown in Figure 6b, the activity of SnRK1 in Ri‐1 and Ri‐3 was approximately 1.25‐ and 1.37‐fold higher than that of wild‐type DJ, respectively. It has been reported that OE of *Arabidopsis SnRK1.1* confers an ABA‐hypersensitive phenotype (Jossier *et al*., 2009). To assess the ABA sensitivity of *OsUbc13*‐RNAi lines, we treated Ri‐1 and Ri‐3 seeds on half‐strength Murashige and Skoog (1/2 MS) medium supplemented with or without 3 μM ABA for 8 d. After ABA treatment, the *OsUbc13*‐RNAi lines had significantly shorter roots and shoots than those of wild‐type DJ, exhibiting hypersensitivity to ABA (Figure S8a,b). Without ABA treatment, the root and shoot lengths of *OsUbc13*‐RNAi lines were similar to DJ, and the roots of Ri‐1 were even longer than DJ (Figure S8a,c). These results demonstrate that silencing *OsUbc13* may increase the activity of OsSnRK1a rather than changing its protein content.

As the two most frequently detected ubiquitination modifications to date, K48‐linked polyubiquitination often leads to the degradation of target protein (Hershko and Ciechanover, 1998; Komander and Rape, 2012; Wickliffe *et al*., 2011), while K63‐linked polyubiquitination is involved in altering the activity of target protein (Chen and Sun, 2009; Li *et al*., 2013; Tang *et al*., 2019; Zhang *et al*., 2019). Ubc13 and its cofactor UEV are unique among E2 ubiquitin‐conjugating enzymes in that they catalyse exclusively the formation of K63‐linked polyubiquitin chains (Lim and Lim, 2011). In tomatoes, Fni3(Ubc13) and Suv (UEV) are reported to together catalyse K63‐specific polyubiquitination (Mural *et al*., 2013). Based on these findings, we investigated whether the ubiquitination of OsSnRK1a is mediated by OsUbc13. We purified OsSnRK1a from the leaves of DJ, *OsUbc13*‐OE (OE17‐2), and *OsUbc13*‐RNAi (Ri‐3) seedlings using anti‐OsSnRK1a polyclonal antibodies. And then, the immunoprecipitated proteins were immunoblotted with two specific ubiquitin antibodies, K48‐ and K63‐Ub chain antibodies (anti‐K48 and anti‐K63). As shown in Figure 6c, the degree of K63‐linked polyubiquitination of immunoprecipitated OsSnRK1a was indistinguishable in OE17‐2 but was visibly attenuated in Ri‐3, relative to those in DJ. The levels of K48‐linked polyubiquitin chains in all materials were very low and did not differ (Figure 6c), suggesting that down‐regulated expression of *OsUbc13* has reduced the specific binding of K63‐linked ubiquitin chains on OsSnRK1a. Taken together, these data show that inhibition of *OsUbc13* expression attenuates K63‐linked polyubiquitination on OsSnRK1a, resulting in the enhanced activity of SnRK1, which in turn improves disease resistance.

### Deubiquitinating enzyme OsOTUB1.1 also physically interacts with OsSnRK1a

**Figure 7:**
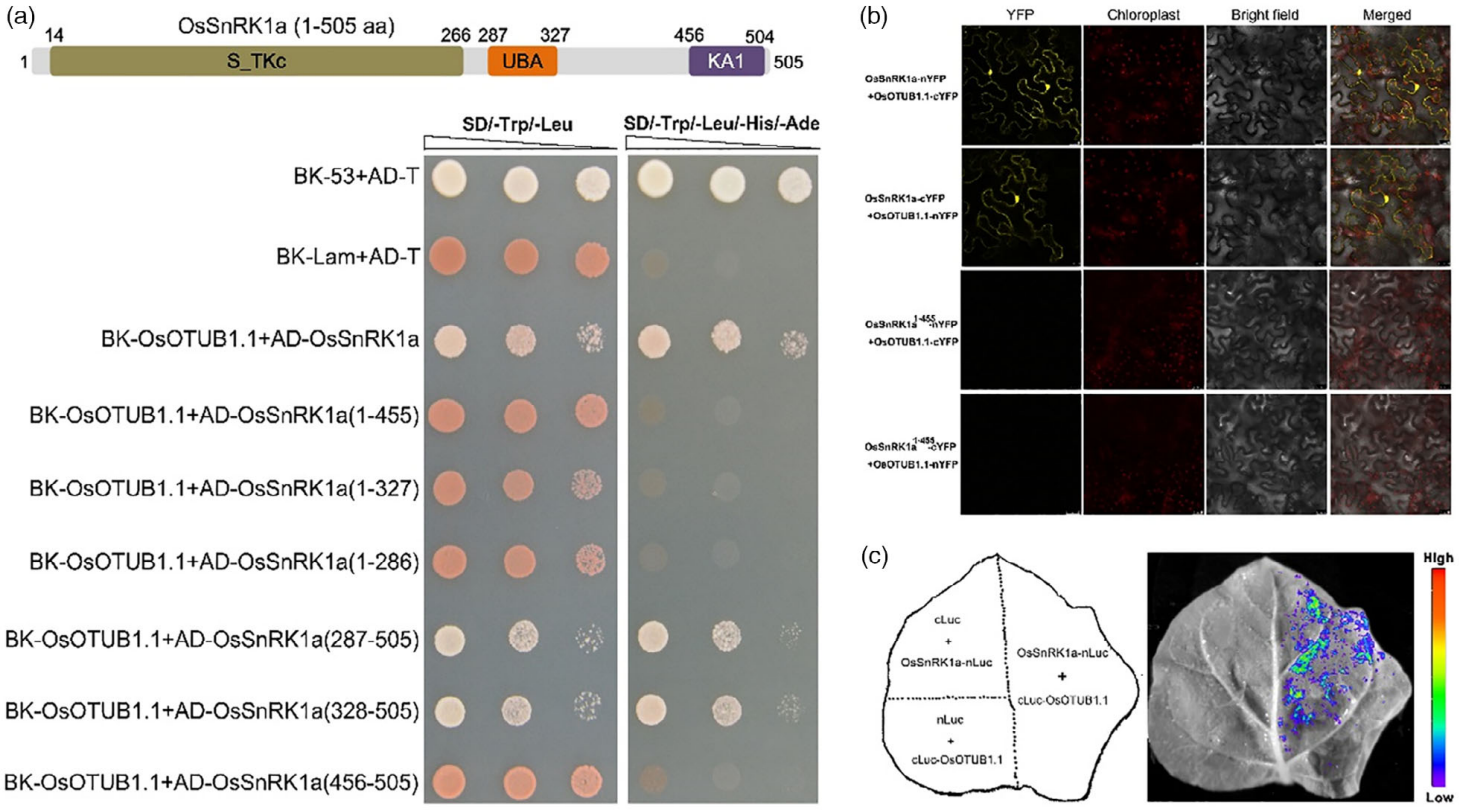
OsOTUB1.1 interacts with OsSnRK1a. (a) Interaction test between OsOTUB1.1 and different truncated OsSnRK1a proteins with specific deletion. Top, the schematic diagram of OsSnRK1a protein. S_TKc, serine/treonine kinase catalytic domain; UBA, ubiquitin‐associated domain; KA1, kinase‐associated 1 domain. Low, the interaction was indicated by the ability of yeast cells to grow on dropout medium lacking Leu, Trp, His, and Ade for 5 days after plating. Yeast cells co‐transformed with AD‐T/BK‐53 or AD‐T/BK‐Lam vectors were used as the positive or negative control, respectively. (b) BiFC assay. Fluorescence was observed in the nuclear compartment of transformed tobacco (*N. benthamiana*) cells, resulting from the complementation of OsSnRK1a‐nYFP+OsOTUB1.1‐cYFP or OsSnRK1a‐cYFP+OsOTUB1.1‐nYFP. No signal was obtained for the negative controls in which OsSnRK1a^1–455^‐nYFP was co‐expressed with OsOTUB1.1‐cYFP, and OsSnRK1a^1–455^‐cYFP was co‐expressed with OsOTUB1.1‐nYFP. YFP signal was detected by confocal microscopy. Scale bars = 25 μM. (c) LCI assay. Agrobacterial strains containing different combinations of plasmids were co‐infiltrated into tobacco leaves. A cooled charge‐coupled imaging apparatus was used to capture the images. No signal was obtained for the negative controls in which OsSnRK1a‐nLuc was co‐expressed with cLuc, and cLuc‐OsOTUB1.1 was co‐expressed with nLuc. The pseudocolor bar indicates the range of luminescence intensity.

In humans, OTUB1, a deubiquitinating enzyme, forms a complex *in vivo* with Ubc13 and prevents ubiquitin transfer (Nakada *et al*., 2010; Wiener *et al*., 2012). OsOTUB1.1 (Os08g0537800), a homologue of TUB1 in rice, can interact with OsUbc13 and cleave both K48‐ and K63‐linked polyubiquitin (Wang *et al*., 2017b). More importantly, rice plants overexpressing *OsOTUB1.1* exhibit leaf necrosis like *OsUbc13*‐RNAi lines (Wang *et al*., 2017b), which prompted us to examine the possibility that OsOTUB1.1 can also interact with OsSnRK1a. As expected, we found an interaction between OsOTUB1.1 and OsSnRK1a in the Y2H system (Figure 7a). To identify the region of OsSnRK1a essential for the interaction with OsOTUB1.1, we fused six truncated OsSnRK1a variants to the prey vector and examined the interaction between these variants and OsOTUB1.1. As shown in Figure 7a, deletion of the C‐terminal amino acid residues 456–505, 328–505, or 287–505 of OsSnRK1a (AD‐OsSnRK1a^1–455^, AD‐OsSnRK1a^1–327^, and AD‐OsSnRK1a^1–286^) completely abolished the OsOTUB1.1‐OsSnRK1a interaction, whereas only retaining the 219 or 178 residues at the C‐terminus of OsSnRK1a that harbour the KA1 domain (AD‐OsSnRK1a^287–505^ and AD‐OsSnRK1a^328–505^) did not affect the OsOTUB1.1‐OsSnRK1a interaction. This result showed that the C‐terminal region of OsSnRK1a is also required for its interaction with OsOTUB1.1. Further mapping revealed that 178 amino acids spanning the C‐terminal KA1 domain (OsSnRK1a^328–505^), rather than just the KA1 domain, are exclusively involved in the OsOTUB1.1‐OsSnRK1a interaction because an OsSnRK1a variant in which the N‐terminal amino acids 1–455 were deleted and only the KA1 domain was retained (AD‐OsSnRK1a^456–505^) could not physically associate with OsOTUB1.1 (Figure 7a). These findings in Y2H assay indicate that the key region of interaction between OsOTUB1.1 and OsSnRK1a is consistent with that of the OsUbc13‐OsSnRK1a interaction.

BiFC assay was performed to determine whether and where OsOTUB1.1 and OsSnRK1a interact in plant cells. The N‐ or C‐terminal of YFP was coupled to OsOTUB1.1 (OsOTUB1.1‐nYFP/cYFP), respectively. And then, these newly constructed recombinant vectors were transiently expressed with OsSnRK1a‐n/cYFP in tobacco leaves. The strong YFP signal was visualized mainly in the OsSnRK1a‐nYFP/OsOTUB1.1‐cYFP and OsSnRK1a‐cYFP/OsOTUB1.1‐nYFP complementation assay in the cytoplasm and nucleus of the transformed cells, but not in the negative controls (Figure 7b). Furthermore, LCI assay was carried out to further confirm the interaction in plants. The cLuc was connected to OsOTUB1.1. OsSnRK1a‐nLuc and cLuc‐OsOTUB1.1 were transiently co‐expressed in tobacco leaves. A strong fluorescence signal was observed in leaves co‐expressing OsSnRK1a‐nLuc and cLuc‐OsOTUB1.1, but not in the leaves expressing plasmids for negative controls (Figure 7c). Together, these results demonstrate that OsOTUB1.1 also interacts with OsSnRK1a in planta.

### *OsOTUB1.* *1*‐OE lines confer enhanced resistance to *Magnaporthe oryzae*, increased SnRK1 activity, and attenuated K63 ubiquitination of OsSnRK1a

**Figure 8:**
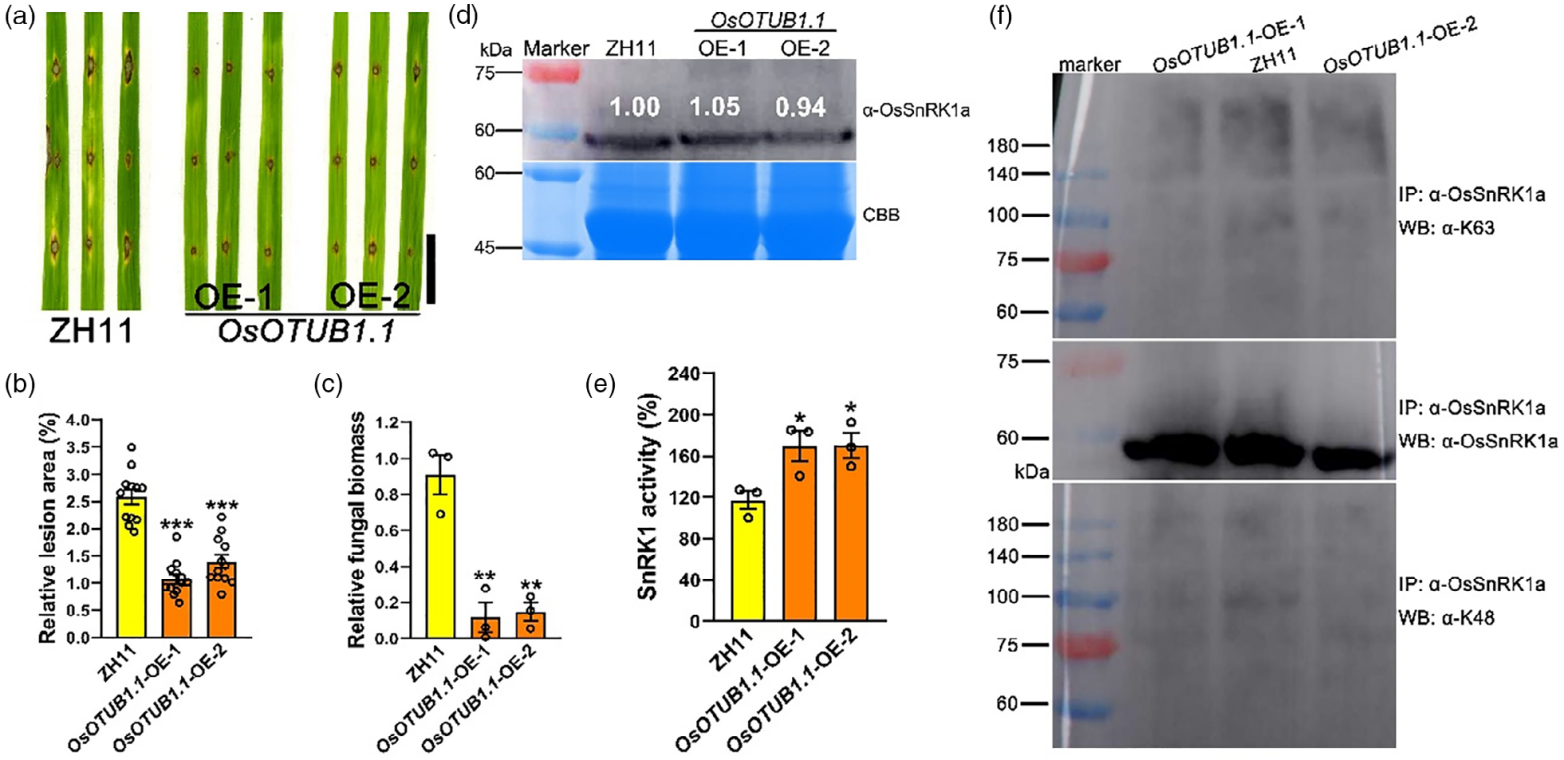
The *OsOTUB1.1*‐OE lines display enhanced resistance to *M. oryzae*. (a) The lesions on ZH11 and *OsOTUB1.1*‐OE leaves at 8 days after punch inoculation with the compatible *M. oryzae* isolate GUY11. Scale bar = 1 cm. (b) Relative lesion area (%) in leaves of (a) indicates significant differences between ZH11 and *OsOTUB1.1*‐OE. Data are shown as means ±SE; *n* = 12 (***P < 0.001; Student's *t*‐test). (c) Relative fungal biomass, measured as *MoPot2* by qRT‐PCR, in leaves of (a) was normalized to *OsUbq* DNA (Park *et al*., 2012). Data are shown as means ± SE; *n* = 3 (**P < 0.01; Student's *t*‐test). (d) The protein level of OsSnRK1a in leaves of ZH11 and *OsOTUB1.1*‐OE plants at 30‐day post‐sowing in soil was detected using anti‐OsSnRK1a. Coomassie brilliant blue (CBB) staining was used as a loading control. Band intensity was calculated by ImageJ software. Experiments were repeated three times with similar results. (e) The SnRK1 kinase activity in leaves of ZH11 and *OsOTUB1.1*‐OE plants at 30‐day post‐sowing in soil. Data are shown as means ±SE; *n* = 3 (*P < 0.05; Student's *t*‐test). (f) *In vivo* polyubiquitination level of OsSnRK1a in ZH11 and *OsOTUB1.1*‐OE lines. Crude protein extracted from seedlings at 30‐day post‐sowing in soil was immunoprecipitated by anti‐OsSnRK1a antibody and detected using antibodies that specifically recognize K48/K63‐polyubiquitin conjugates (anti‐K48 and K63) and anti‐OsSnRK1a antibody. Experiments were repeated three times with similar results.

Considering that OsOTUB1.1 can also interact with OsSnRK1a, we wondered whether OsOTUB1.1 is involved in rice disease resistance. First, we obtained two independent *OsOTUB1.1*‐ OE lines (*OsOTUB1.1*‐OE‐1 and *OsOTUB1.1*‐OE‐2, Figure S9a) in the background of Zhonghua 11 (ZH11, Wang *et al*., 2017b). Then, we inoculated them with *M. oryzae* isolate GUY11 by the punch method, and we found that the two *OsOTUB1.1*‐OE lines both increased disease resistance to *M. oryzae*, which showed a smaller lesion area and less fungal biomass than the wild‐type ZH11 (Figure 8a–c). To ascertain whether enhanced disease resistance response stemmed from elevated expression of PR genes, we examined their expression by qRT‐PCR. As components of JA signalling or biosynthesis, *WRKY45*, *JAMyb*, *JAZ8*, *PBZ1*, and *AOS2* genes (Sathe *et al*., 2019) were significantly up‐regulated in the two *OsOTUB1.1*‐OE lines compared with ZH11 (Figure S9b–f). Most of the SA‐signalling genes, *PAL3*, *PR1a*, *PR3*, *PR4*, *PR10*, *PR4*, and *PR10* were highly expressed in *OsOTUB1.1*‐OE lines compared with ZH11 (Figure S9g–m). Collectively, these data suggest that, similar to the *OsUbc13*‐RNAi lines, the *OsOTUB1.1*‐OE lines are likely to elevate disease resistance by activating SA and JA signalling pathways in rice.

Although in a different background, OE of *OsOTUB1.1* also showed identical phenotypes to those of plants overexpressing *OsSnRK1a*, in terms of the resistance to *M. oryzae*, plant height, and the number of tillers per plant (Filipe *et al*., 2018; Wang *et al*., 2017b). We speculated that there are two possibilities for the appearance of the above phenotypes, the protein level or the activity of OsSnRK1a was increased in the *OsOTUB1.1*‐OE line. Because there was no difference in the abundance of the *OsSnRK1a* transcript between ZH11 and *OsOTUB1.1*‐OE lines (Figure S9n), so we assayed the protein contents of OsSnRK1a using anti‐OsSnRK1a antibody. As shown in Figure 8d, the level of OsSnRK1a protein was not significantly increased in the *OsOTUB1.1*‐OE line compared with wild‐type ZH11, ruling out one of these possibilities. The other possibility was investigated by measuring SnRK1 activity in seedlings of *OsOTUB1.1*‐RNAi lines. As shown in Figure 8e, the SnRK1activity of *OsOTUB1.1*‐OE‐1/2 was approximately 1.45‐fold higher than that of wild‐type ZH11. And then, the immunoprecipitated OsSnRK1a proteins from ZH11 and the two *OsOTUB1.1*‐OE seedlings were immunoblotted using anti‐K48 and anti‐K63 antibodies. The degree of K63‐linked polyubiquitination of OsSnRK1a was visibly attenuated in *OsOTUB1.1*‐OE lines. The levels of K48‐linked polyubiquitin chains in all materials were very low and did not differ (Figure 8f). Collectively, the above results prove that, like inhibiting *OsUbc13*, overexpressing *OsOTUB1.1* reduces the K63‐linked ubiquitin chains on OsSnRK1a, enhances SnRK1 activity, and in turn improves rice blast resistance.

### OsUbc13 affects rice blast resistance partially dependent on OsSnRK1a

**Figure 9:**
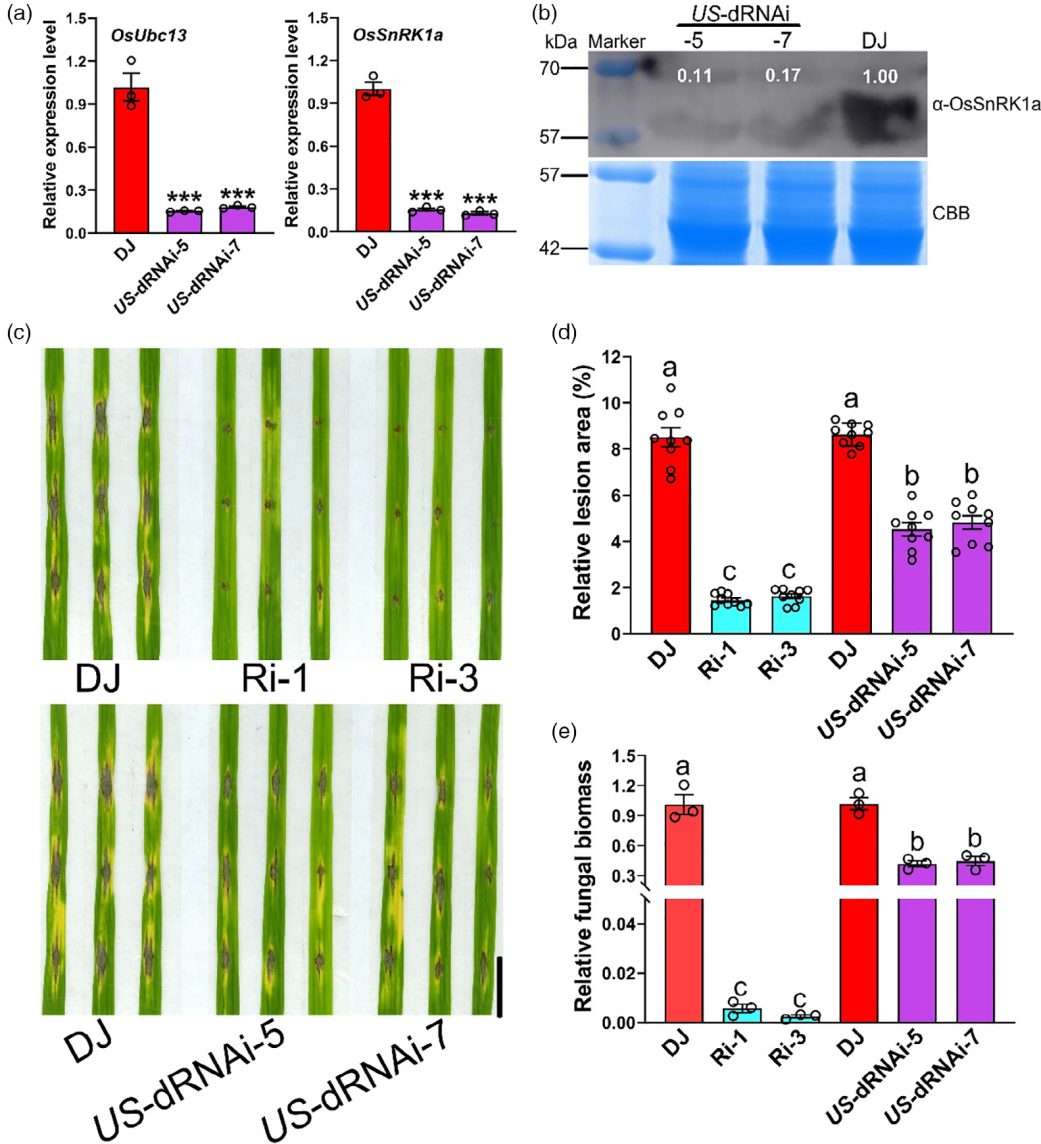
Repression of *OsSnRK1a* partially reduces the higher resistance to *M. oryzae* in *OsUbc13*‐RNAi line (Ri‐3). (a) Expression levels of OsUbc13 and OsSnRK1a in DJ and *US*‐dRNAi plants. The double RNA interference materials named *US*‐dRNAi were obtained from the re‐interference of *OsSnRK1a* in the *OsUbc13*‐RNAi homozygous line (Ri‐3). *OsActin1* gene was used as an internal control. Data are shown as means ±SE; *n* = 3 (***P < 0.001; Student's *t*‐test). (b) The protein level of OsSnRK1a in leaves of DJ and *US*‐dRNAi plants at 30‐day post‐sowing in soil was detected using anti‐OsSnRK1a. Coomassie brilliant blue (CBB) staining was used as a loading control. Experiments were repeated two times with similar results. (c) The lesions on DJ, OsUbc13‐RNAi, and *US*‐dRNAi leaves at 8 days after punch inoculation with the compatible *M. oryzae* isolate GUY11. Scale bar = 1 cm. (d) Relative lesion area (%) in leaves of (b) indicates significant differences between DJ, OsUbc13‐RNAi, and *US*‐dRNAi. Data are shown as means ±SE; *n* = 9. Significant difference was determined by ANOVA; values with different letters indicate a significant difference from each other (*P* < 0.05). (e) Relative fungal biomass, measured as *MoPot2* by qRT‐PCR, in leaves of (b) was normalized to *OsUbq* DNA (reference). Data are shown as means ±SE; *n* = 3 (**P < 0.01; Student's *t*‐test).

To provide genetic evidence for the regulation of OsSnRK1a by OsUbc13, we obtained double RNAi lines by transforming the *OsSnRK1a*‐RNAi plasmids into one *OsUbc13*‐RNAi line (Ri‐3), namely *US*‐dRNAi. Two independent transgenic lines, *US*‐dRNAi‐5 and *US*‐dRNAi‐7, were identified. The qRT‐PCR confirmed the decrease in target transcript abundance in the *US*‐dRNAi lines (Figure 9a). OsSnRK1a protein levels in the *US*‐dRNAi lines were also significantly lower than that in DJ (Figure 9b). Then, we inoculated them with *M. oryzae* isolate GUY11 by the punch method, and we found that down‐regulation of *OsSnRK1a* in *US*‐dRNAi plants resulted in decreased disease resistance to *M. oryzae* to a level intermediate to those of *OsUbc13*‐RNAi lines and wild‐type DJ (Figure 9c). Statistically, the two *US*‐dRNAi lines displayed smaller lesion areas and less fungal biomass compared with wild‐type DJ, but these did not reach the levels of *OsUbc13*‐RNAi (Figure 9d,e). Thus, genetically decreasing *OsSnRK1a* expression partially suppressed rice blast resistance phenotypes of *OsUbc13*‐RNAi lines in rice.

## Discussion

Ubiquitin‐conjugating enzyme (Ubc) 13 plays a key role for the regulation of both innate and adaptive immune responses in mammalian immune systems (Jiang and Chen, 2011). Consequently, the Ubc13 protein has become a target of pathogens in suppressing host immunity (Sanada *et al*., 2012). Compared with its well‐studied mammalian ortholog, the function of Ubc13 in plants is less explored. Fni3, the homologue of mammalian Ubc13 in tomato, is solely responsible for catalysing Lys63(K63)‐linked ubiquitination and has been reported to regulate HR‐related PCD and plays a role in ETI (Mural *et al*., 2013). Arabidopsis *Ubc13* differentially regulates two PCD pathways in responses to pathogen and low‐temperature stress (Wang *et al*., 2019), suggesting that Ubc13 has a conserved function to regulate immune responses in different plants. In addition to immunity, plant Ubc13 (Li and Schmidt, 2010; Wen *et al*., 2006, 2014; Yin *et al*., 2007) and Ubc13‐mediated K63 polyubiquitination (Johnson and Vert, 2016; Martins *et al*., 2015; Romero‐Barrios and Vert, 2018; Tomanov *et al*., 2014) have been implicated in numerous biological processes.

The rice genome only encodes one Ubc13 protein named OsUbc13. However, its function in rice immunity and disease resistance has not been fully understood. In the present study, we first found that silencing the *OsUbc13* gene in the DongJin (DJ) background triggers a series of immune responses, including typical necrotic lesions, cell death, and ROS burst (Figure 1). Additionally, two key agronomic traits including tiller number and grain number per panicle were adversely affected in *OsUbc13*‐RNAi plants (Figure S1b,c), which is consistent with perturbation of the *OsUbc13* gene in the Zhonghua11 (ZH11) background by another research group (Wang *et al*., 2017b). Next, through personal communication with the first author of this article (Wang *et al*., 2017b), we learned that disrupting the *OsUbc13* gene in ZH11 also leads to a lesion‐mimic phenotype, further consolidating our results. We also tried to knockout *OsUbc13* using the CRISPR/Cas9 method, but all the transgenic lines of T_0_ generation died at the seedling stage. Moreover, it has been reported that *OsUbc13* is a candidate housekeeping gene (Zang *et al*., 2012), so it is possible that the knockout of *OsUbc13* is lethal and the normal growth of rice must depend on a functional OsUbc13.

In plants, for example, enhanced defence is often accompanied by a ‘syndrome’ of compromised morphology such as growth retardation, male sterility, and reduced plant size. In other words, such compromised morphology often results in immunity activation as observed in transgenic rice overexpressing *OsWRKY45* and *Ideal Plant Architecture1* , knockdown of *miR156*, and knockout of *OsDOF11* or *OsALDH2B1*, which is often referred to as trade‐offs between growth and defence (Goto *et al*., 2015; Ke *et al*., 2020; Li *et al*., 2016; Wu *et al*., 2018; Yang *et al*., 2012). So, the reduction of tiller numbers and grain numbers per panicle in the *OsUbc13*‐RNAi lines was probably caused by autoactivation of immune responses. *Arabidopsis ubc13* double mutant has similar immune responses with the *OsUbc13*‐RNAi lines. However, these responses of *ubc13* are induced by low temperature, which only appears when grown at 16 °C, not at 22 °C (Wang *et al*., 2019). In addition to ROS bursts and cell death, many lesion‐mimic mutants exhibit enhanced disease resistance (Ma *et al*., 2019; Qiao *et al*., 2010; Qiu *et al*., 2021; Yamanouchi *et al*., 2002). Unlike typical lesion‐mimic mutants, *Arabidopsis ubc13* double mutant did not enhance disease resistance against virulent bacterial and fungal pathogens, but diminished HR and compromised ETI against avirulent bacterial pathogens (Wang *et al*., 2019). Unexpectedly, in our study, the *OsUbc13*‐RNAi lines were found to show enhanced resistance to both fungal and bacterial pathogens, accompanied by up‐regulated expression of defence‐related genes and elevated levels of defence hormones (Figures 2 and 3). Although both homologous Ubc13s are involved in autoimmune responses, their contributions to disease resistance in *Arabidopsis* (dicot) and rice (monocot) are diametrically opposed, demonstrating the complexity of Ubc13's regulatory mechanisms on immune responses. Similarly, there are differences in the NPR1‐mediated PCD and resistance in *Arabidopsis* and rice. In *Arabidopsis*, AtNPR1 is a negative regulator of PCD (Fu *et al*., 2012; Furniss and Spoel, 2015). By contrast, OsNPR1 positively regulates PCD in rice (Bai *et al*., 2011; Chern *et al*., 2005; Liu *et al*., 2017; Yuan *et al*., 2007).

Sucrose non‐fermenting 1 (SNF1)‐related kinase 1 (SnRK1) is a central energy sensor kinase in plants that is functionally and evolutionarily conserved with SNF1 in yeast and AMP‐activated kinase (AMPK) in animals (Baena‐González and Lunn, 2020; Broeckx *et al*., 2016; Crepin and Rolland, 2019; Crozet *et al*., 2014). The eukaryotic AMPK/SNF1/SnRK1 protein kinases typically function as heterotrimeric complexes composed of one α‐catalytic subunit and two regulatory subunits, β and γ (Broeckx *et al*., 2016; Crozet *et al*., 2014). Several recent studies have shown that SnRK1 in plants is involved in various metabolic pathways (Cai, 2020; Hu *et al*., 2022; Liang *et al*., 2021; Wang *et al*., 2020, 2021; Zhang *et al*., 2020c). Notably, increasing evidence indicates that SnRK1 also plays an important role in plant biotic interactions (Han *et al*., 2020; Hao *et al*., 2003; Jiang *et al*., 2020; Shen *et al*., 2011; Shen and Hanley‐Bowdoin, 2021). In rice, the three genes encoding α‐catalytic subunit of SnRK1 are classified into two subfamilies, namely *OsSnRK1a* (*OSK1*) and *OsSnRK1b* (*OSK24* and *OSK35*). Among them, OSK35 positively regulates defence against *M*. *oryzae* and *Xoo* (Kim *et al*., 2015). Recently, OsSnRK1a has been reported to confer broad‐spectrum disease resistance and act as a master switch that regulates growth‐immunity trade‐offs in rice (Filipe *et al*., 2018), but the mechanisms of OsSnRK1a‐mediated immune regulation remain unknown.

Wang *et al*., (2014) identified that OsSnRK1a may be a potential interacting protein of OsUbc13. Based on Y2H, LCI, Co‐IP, and BiFC analysis, we confirmed that OsUbc13 associates with OsSnRK1a *in vivo* (Figure 4a–d). Conventionally, ubiquitin‐conjugating enzymes (Ubcs or E2s) are thought to interact primarily with ubiquitin ligases (E3s). Nevertheless, E2 enzymes have been shown in several cases to interact with substrate proteins of ubiquitination (Laine *et al*., 2006; Mural *et al*., 2013; Shembade *et al*., 2007). *In vitro* pull‐down assay showed that the interaction between OsUbc13 and OsSnRK1a is direct (Figure 4e). Coincidentally, a previous literature also reported that Fni3, an ortholog of OsUbc13 in tomato (Figure S4), directly binds a protein kinase Fen and catalyses exclusively K63‐linked ubiquitination (Mural *et al*., 2013). Further analysis revealed that 178 amino acids containing the C‐terminal KA1 domain of OsSnRK1a (OsSnRK1a^328–505^), rather than just the KA1 domain, are essential for the OsUbc13‐OsSnRK1a interaction (Figure 5a). Substitution of Cys‐89 (the active site for ubiquitin thioester formation) in Fni3 and OsUbc13 with Gly essentially abolishes their interactions with Fen or OsSnRK1a (Mural *et al*., 2013; Figure 5b). However, as a protein kinase (PK), substitution of Lys‐43 (ATP binding site for PK) or Lys‐139 (catalytic site for PK) in OsSnRK1a (Cho *et al*., 2012) does not affect its interaction with OsUbc13 (Figure 5b), indicating that their binding may be more dependent on the ubiquitin‐conjugating enzyme activity of OsUbc13 rather than the kinase activity of OsSnRK1a.

Formation of K63‐linked polyubiquitination requires specific dimeric complex formed by Ubc13 and another Ubc‐E2 variant (UEV) protein because Ubc13 lacks active cysteine residues found in other E2s (Broomfield *et al*., 1998; Hofmann and Pickart, 1999; Romero‐Barrios and Vert, 2018). In our previous study, it was found that OsUEV1B, an Ubc enzyme variant protein, interacts with OsUbc13 and is required for phosphate homeostasis in rice (Liu *et al*., 2021). As expected, OsUEV1B was also able to bind to OsSnRK1a in the Y2H system (Figure 5c). However, unlike the *OsUbc13*‐RNAi lines, the *osuev1b* mutant did not exhibit stronger blast resistance than wild‐type DJ (Figure S5), probably due to functional redundancy among the four members of the OsUEV1 family (named OsUEV1A‐D) in rice, whereas OsUbc13 is unique (Wang *et al*., 2017a; Zang *et al*., 2012). These findings imply that the enhanced disease resistance after interfering with *OsUbc13* is likely related to the altered ubiquitination level of OsSnRK1a.

Considering that the effects of down‐expression of *OsUbc13* or over‐expression of *OsSnRK1a* on rice disease resistance are consistent, we speculate that the protein level or activity of OsSnRK1a will be elevated in *OsUbc13*‐RNAi lines. By western blot analysis, we found that the protein content of OsSnRK1a did not change significantly in both the *OsUbc13* interference and OE lines, and the same was true at the transcriptional level (Figures 6a and S7). *OsSnRK1a* encodes the α catalytic subunit of SnRK1, and it has been reported that OE of *Arabidopsis SnRK1.1* confers higher SnRK1 activity an ABA‐hypersensitive phenotype (Jossier *et al*., 2009). Excitingly, in the present study, SnRK1 activity (as indicated by OsSnRK1a activity), was significantly improved in the *OsUbc13*‐RNAi lines, and it was accompanied by increased ABA sensitivity (Figures 6b and S8). Unlike conventional K48‐linked ubiquitination that mainly serves as a signal for 26S proteasome‐mediated degradation of substrate proteins, K63‐linked polyubiquitination has been shown to play nonproteolytic, regulatory roles in various cellular processes (Bhoj and Chen, 2009; Duncan *et al*., 2006; Fisk and Yaffe, 1999; Lauwers *et al*., 2009; Martinez‐Forero *et al*., 2009). So far, Ubc13 is the only known E2 that mediates the polyubiquitination linked by K63, and K63‐linked polyubiquitination, similar to phosphorylation or sumoylation, changes the activities of target proteins (Pickart, 2001). In recent years, there have been such reports. For example, HECTD3, a new E3 ubiquitin ligase, interacts with caspase‐8 death effector domains and ubiquitinates caspase‐8 with K63‐linked polyubiquitin chains that do not target caspase‐8 for degradation but decrease the caspase‐8 activation (Li *et al*., 2013). K63‐linked ubiquitination suppresses RIPK1 kinase activity to regulate cell death during embryogenesis and inflammation (Tang *et al*., 2019; Zhang *et al*., 2019). Using two specific ubiquitin antibodies, K48‐ and K63‐Ub chain antibodies (anti‐K48 and anti‐K63), we found the degree of K63‐linked polyubiquitination for OsSnRK1a in Ri‐3 (one *OsUbc13*‐RNAi line) was visibly attenuated, relative to those in DJ. The levels of K48‐linked polyubiquitin chains did not differ (Figure 6c). Taken together, these data support our hypothesis that down‐regulation of *OsUbc13* enhances the kinase activity of OsSnRK1a, most likely by attenuating K63‐linked polyubiquitination of OsSnRK1a. Substitution of the active ubiquitination site (K276R) in AtPIP2;1 significantly reduces its ubiquitination (Chen *et al*., 2021). According to previous research on rice ubiquitome (Xie *et al*., 2015), there are two predicted ubiquitinated lysine motifs (EK^ub^) in the KA1 domain of OsSnRK1a, at positions 458 and 476. Further LC–MS/MS and point mutation analysis would be worthwhile to determine whether these two lysine residues (K458 and K476) are active ubiquitination sites and their effects on ubiquitination and activity of OsSnRK1a.

Surprisingly, our further study revealed that OsOTUB1.1, a deubiquitinase known to interact with OsUbc13 for cleaving both K48‐ and K63‐linked polyubiquitin, also binds to OsSnRK1a, and even the key regions required for their bindings are consistent (Figure 7). Rice plants overexpressing *OsOTUB1.1* exhibit similar phenotypes with *OsUbc13*‐RNAi lines, including leaf necrosis (Wang *et al*., 2017b). Phenotypic identification and qRT‐PCR results showed that OE of *OsOTUB1.1* (Figure S9a) also significantly increased rice resistance to *M. oryzae* and the expression of defence‐related genes (Figures 8a–c and S9b–m). Likewise, OE of *OsOTUB1.1* increased SnRK1 activity and reduced the K63‐linked polyubiquitination of OsSnRK1a, also having little effect on OsSnRK1a at the transcriptional and translational levels (Figures 8d–f and S9n). These results further suggest that K63‐linked polyubiquitination inhibits the activity of OsSnRK1a, which can be alleviated by down‐regulating *OsUbc13* or up‐regulating *OsOTUB1.1*. Further investigation is warranted to determine whether OsUbc13 and OsOTUB1.1 competitively interact with OsSnRK1a and their relationship in rice immunity.

**Figure 10:**
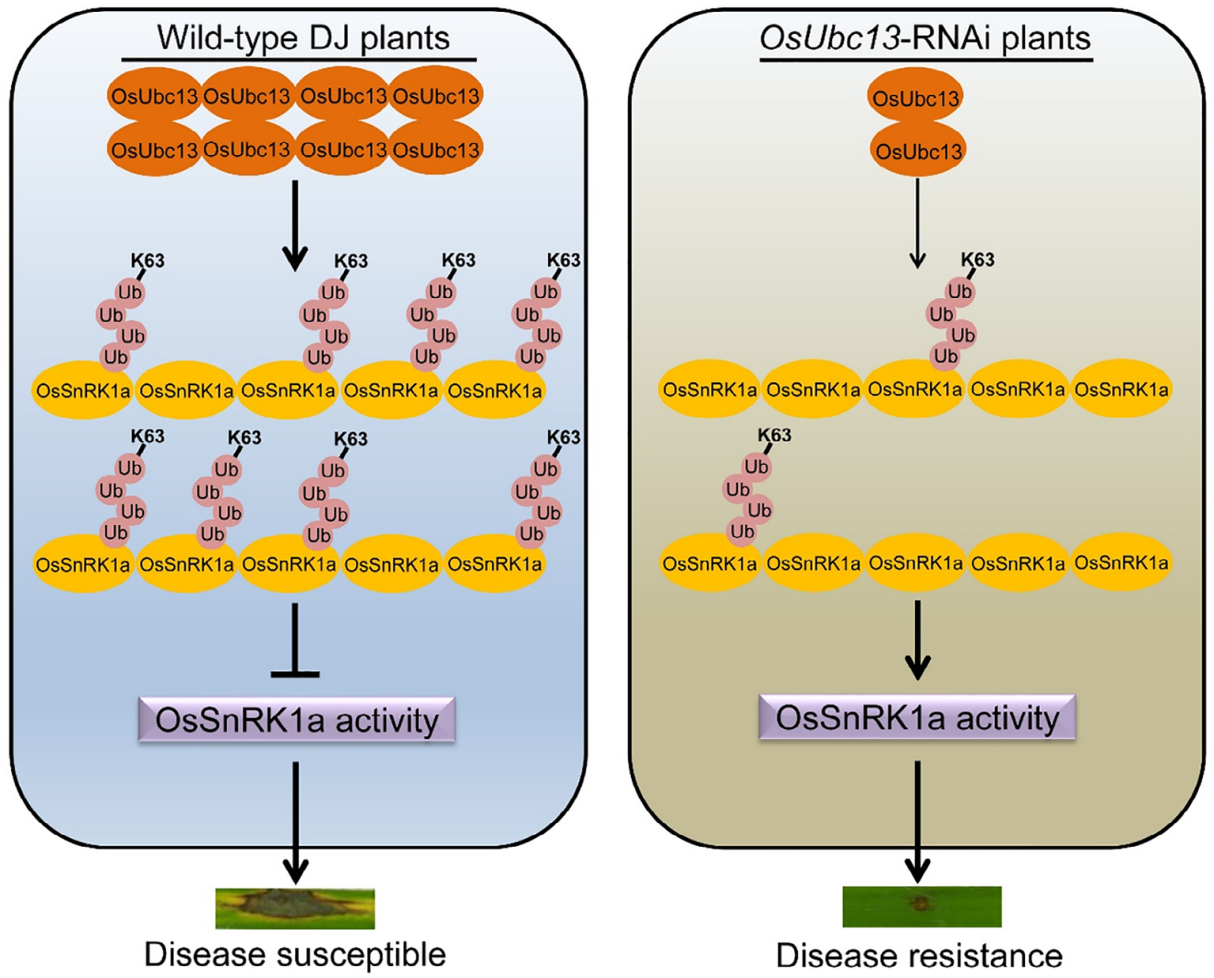
A proposed model for OsUbc13‐mediated blast resistance. In wild‐type DJ plants, OsUbc13 interacts with OsSnRK1a, resulting in high levels of K63‐linked polyubiquitination on OsSnRK1a, inhibition of its activity, and becoming susceptible to *M. oryzae*. In the *OsUbc13*‐RNAi plants, a small amount of OsUbc13 does not allow more K63‐linked polyubiquitination on OsSnRK1a, leading to increased activity of OsSnRK1a and enhanced resistance to *M. oryzae*.

After re‐interfering with *OsSnRK1a* in one *OsUbc13*‐RNAi line (Ri‐3), the *M. oryzae* resistance of the double‐interfering materials (name *US*‐dRNAi) was reduced to an intermediate level between the wild‐type DJ and *OsUbc13*‐RNAi (Figure 9), providing genetic evidence that OsUbc13 negatively regulates blast resistance partially via OsSnRK1a. We present a possible working model for OsUbc13‐OsSnRK1a module based on our results and those of previous reports (Figure 10). In wild‐type DJ plants, K63‐linked polyubiquitination of OsSnRK1a is maintained at high levels by interacting with OsUbc13, resulting in inhibition of its activity and, thus, becoming susceptible to *M. oryzae*. However, in the *OsUbc13*‐RNAi plants, K63‐linked polyubiquitination of OsSnRK1a is attenuated, increasing its activity, which in turn enhances resistance to *M. oryzae*. A major task in understanding Ubc13‐mediated K63 polyubiquitination in plants and its biological relevance is to identify its cognate E3s. So far, in *Arabidopsis*, only one RING domain‐containing protein, RGLG2, and another F‐box protein, CPR1, have been identified to interact with Ubc13 and function in apical dominance and immunity (Wang *et al*., 2019; Yin *et al*., 2007). Yeast two‐hybrid analyses using the U‐box‐domain regions of armadillo (ARM)‐U‐box E3 Ub‐ligases and the Ub‐conjugating (UBC) domains of E2s showed that, among 40 rice E2s, 11 E2s accounted for 70% of the interactions with 17 ARM‐U‐box E3s. But none of the ARM‐U‐box E3 had been identified to interact with OsUbc13 (Bae and Kim, 2014). In this study, we have identified OsUbc13 capable of direct interaction with OsSnRK1a *in vitro*, indicating that ubiquitination of the latter by the former may be independent of additional E3, which needs to be confirmed by *in vitro* ubiquitination assay. Silencing of *OsSnRK1a* in the Ri‐3 only partially rescued the phenotype of blast resistance. Identifying other proteins that interact with OsUbc13 will help us to understand OsUbc13‐mediated immunity responses and disease resistance in rice more comprehensively.

## Materials and methods

### Plant materials and growth conditions

The *OsUbc13*‐RNAi and *OsUbc13*‐OE transgenic plants, as well as previously obtained *osuev1b* and *osvdac1* mutants (Liu *et al*., 2021) were generated in the background of *Oryza sativa* ssp. *japonica* cv. DongJin (DJ). The *OsOTUB1.1*‐OE lines in the background of *Oryza sativa* ssp. *japonica* cv. Zhonghua11 (ZH11) were kindly provided by Prof. Xiangdong Fu and Dr. Shuansuo Wang (Institute of Genetics and Developmental Biology, Chinese Academy of Sciences). The double RNA interference materials named *US*‐dRNAi were obtained from the re‐interference of *OsSnRK1a* in the *OsUbc13*‐RNAi homozygous line (Ri‐3). For disease and other phenotype characterizations, the sterilized rice seeds were germinated on 1/2 Murashige & Skoog (MS) medium. Seedlings were grown in a greenhouse under 12‐h/12‐h days (30 °C)/nights (25 °C) photoperiod at a photon density of approximately 200 mmol/m^2^/s and approximately 60% humidity, or in the experimental field of Fujian Agriculture and Forestry University (Sanming, China).

### Expression constructs and their transformation in plants

The RNAi constructs were obtained by subcloning a fragment (~200 bp) from the coding sequence (CDS) of *OsUbc13 or OsSnRK1a* into the pTCK303 vector (Wang *et al*., 2004) in the sense and antisense orientations, respectively. The full‐length CDS of *OsUbc13* (462 bp) was cloned into the modified pCAMBIA1300 vector (target gene driven by the maize Ub gene promoter) to generate OE constructs. The resulting constructs were transformed into DJ or Ri‐3 by *Agrobacterium tumefaciens*‐mediated transformation. The transformants were screened by PCR amplification using primers specific for *HYG* or *G148*. The primers used are listed in Table S1.

### RNA extraction and quantitative reverse transcription PCR analysis

RNA extraction and quantitative real‐time PCR analysis were performed as previously described (Liu *et al*., 2016). Briefly, fresh plant tissues were harvested and immediately ground into a fine powder in liquid nitrogen. Total RNA was extracted using TRIzol reagent (Invitrogen) according to the manufacturer's instructions. The DNase‐treated RNA was reverse transcribed using SuperScript III reverse transcriptase (Invitrogen) according to the manufacturer's instructions. Quantitative reverse transcription PCR (qRT‐PCR) was performed in an optical 96‐well plate using SYBR Premix Ex Taq (TaKaRa) and the CFX96 Real‐Time PCR Detection System (Bio‐Rad). The PCR thermal cycling protocol was 95 °C for 10 s, followed by 40 cycles at 95 °C for 5 s and 60 °C for 30 s. *OsActin1* gene was used as the internal reference, and data analyses were performed using the 2^−ΔΔCt^ method as described previously (Livak and Schmittgen, 2001).

### DAB staining for H_2_O_2_ in leaves

3,3′‐Diaminobenzidine (DAB, Sigma‐Aldrich) staining was performed following a published method with some modifications (Liu *et al*., 2018). Briefly, leaves (second from the top) at 30‐day post‐sowing in soil were detached and immersed in 1% DAB solution in HCl‐acidified (pH 3.8). After 30 min under vacuum, the samples were incubated at room temperature for 24 h in the dark. The samples were then bleached by boiling in ethanol to remove the chlorophyll and reveal the brown spots, which are indicative of the reaction of DAB with H_2_O_2_. The samples were observed and imaged under a scanner.

### Measurement of H_2_O_2_ contents

H_2_O_2_ contents were measured as previously described with some modifications (Liu *et al*., 2018). Briefly, leaves (second from the top) at 30‐day post‐sowing in soil were used to measure H_2_O_2_ contents. The contents were measured spectrophotometrically after reaction with potassium iodide (KI). The reaction mixture consisted of 0.5 mL of 0.1% trichloroacetic acid (TCA), leaf extract supernatant, 0.5 mL of 100 mM potassium phosphate buffer (pH 7.8) and 1 mL reagent (1 M KI, w/v in fresh double‐distilled water). The blank control consisted of 1 mL 0.1% TCA and 1 mL KI in the absence of leaf extract. After 1 h of reaction in darkness, the absorbance was measured at 390 nm. The amount of H_2_O_2_ was calculated using a standard curve prepared with known concentrations of H_2_O_2_.

### Measurement of reactive oxygen species burst

ROS burst was measured following PAMP treatment (flg22 and chitin) as previously described (Liu *et al*., 2017; Park *et al*., 2012; Schwacke and Hager, 1992). Briefly, leaves (second from the top) at 12‐day post‐sowing in nutrient solution were punched into disks (0.25 cm^2^), which were submerged in distilled water overnight. Three disks per sample were then placed in a 1.5‐mL microcentrifuge tube containing 100 mL luminol (Sigma‐Aldrich), 1 mL horseradish peroxidase (Sigma‐Aldrich), and 100 nM flg22 (Phytotech) or 8 nM hexa‐N‐acetyl‐chitohexaose (APExBIO); distilled water was used as a control. The luminescence was immediately measured at 10‐s intervals over a period of 20 min or 41 min in a Glomax 20/20 luminometer (Promega). Three biological replications (three disks for each replication) were performed for each sample of the three treatments.

### Measurement of malondialdehyde and photosynthetic pigment

The seedlings at 30‐day post‐sowing in soil were used to measure MDA and photosynthetic pigment. The contents of chlorophyll a, chlorophyll b, and carotenoids were determined according to He *et al*. (2018). The contents of malonaldehyde (MDA) were measured following the manufacturer's instructions (Nanjing Jiancheng Bioengineering Institute, Nanjing, China).

### Pathogen infection

To evaluate rice blast disease resistance, the clipped leaves (second from the top) or the whole seedlings at 20‐day post‐sowing in nutrient solution were inoculated with *M. oryzae* strain GUY11 (Li *et al*., 2017; Liu *et al*., 2017). GUY11 was gown on complete agar medium for 2 weeks before producing spores. Spores were collected via flooding of the fungal agar cultures with sterile water, and the spore concentration in the suspension was adjusted to 1 × 10^5^ conidia/mL before inoculation. For the punch method, we dip 5 μL spore suspension for each drop using a pipette at 3 spots on each leaf which was wounded with a mouse ear punch and then put them in a culture dish that contains 0.1% 6‐benzylaminopurine (6‐BA) sterile water to keep moist (Ono *et al*., 2001; Park *et al*., 2012). For spraying method, the whole seedlings were sprayed with spore suspension. The inoculated leaves or seedlings were transferred to a growth chamber with a photoperiod of 12 h light and 12 h dark, 28 °C, and 80% relative humidity, and lesion areas were measured using a scanner and ImageJ software after inoculation for 8 days. Relative fungal DNA amount was calculated using the threshold cycle value (C_t_) of *M. oryzae* Pot2 DNA against the C_t_ of rice genomic ubiquitin DNA (Park *et al*., 2012).

To evaluate bacterial blight disease, rice plants were inoculated with *Xoo* by the leaf‐clipping method as previously described (Liu *et al*., 2017). The PXO99 strain was separately suspended in distilled water and adjusted to 10^9^ viable cells/mL (OD_600_ = 1). Scissors were dipped into the bacterial suspensions and then used to remove the distal tips (5 cm) of flag leaves. At least three individual plants and three tillers of each plant were inoculated. The infected plants were grown in a greenhouse under 12‐h/12‐h days (30 °C)/nights (25 °C) photoperiod at a photon density of approximately 200 mmol/m^2^/s and approximately 60% humidity. Disease was scored by measuring the lesion length at 18 days post‐inoculation.

### Plant hormone analysis

The quantification of plant hormones was performed as previously described (Dobrev and Vankova, 2012; Liu *et al*., 2020). In brief, 3 g of plant leaves at 30 days after sowing in soil were rapidly frozen in liquid nitrogen and homogenized into a powder, and the powder was extracted with 1 mL methanol containing 20% water at 4 °C for 12 h. The extract was centrifuged at 12000 g under 4 °C for 15 min. The supernatant was collected and evaporated to dryness under nitrogen gas stream and reconstituted in 100 mL of acetonitrile containing 5% water. The solution was centrifuged, and the supernatant was collected for analysis using an LC‐ESI‐MS/MS system (HPLC, Shim‐pack UFLC SHIMADZU CBM30A system; MS, Applied Biosystems 4500 Q TRAP). The experiments were performed by Wuhan Metware Biotechnology Co., Ltd (Wuhan, China).

### Y2H assay

The full‐length CDS of *OsUbc13, OsUbc13* ^*C89G*^ and *OsOTUB1.1* was fused to pGBKT7 (Clontech) to generate bait vectors (BK‐OsUbc13 and BK‐OsOTUB1.1) that contain the Gal4 DNA‐BD. Full‐length CDS of *OsHRLI*, *OsCPI*, *OsYchF1*, *OsSnRK1a*, *OsSnRK1a* ^*K43M*^, and *OsSnRK1a* ^*K139R*^ was inserted into pGADT7 (Clontech) to produce prey vectors (AD‐OsHRLI, AD‐OsCPI, AD‐OsYchF1, AD‐OsSnRK1a, AD‐OsSnRK1a^K43M^, and OsSnRK1a^K139R^) with the Gal4 DNA‐AD. To identify specific regions critical for the interactions, multiple truncated OsSnRK1a sequences were ligated with pGADT7. Yeast two‐hybrid assays were performed as described previously (Liu *et al*., 2021). The bait and prey vectors were co‐transformed into the yeast strain AH109 and physical interactions were indicated by the ability of cells to grow on a dropout medium lacking Leu, Trp, His, and Ade for 5 days after plating.

### Luciferase complementation imaging assay

LCI assay was performed as previously described (Chen *et al*., 2008; Liu *et al*., 2021). The pCAMBIA1300‐nLuc and pCAMBIA1300‐cLuc vectors were used for this analysis. The cLuc‐OsUbc13, cLuc‐OsOTUB1.1, and OsSnRK1a‐nLuc constructs were obtained by enzyme digestion and ligation or by seamless cloning. Different pairs of constructs were co‐transformed into wild‐type tobacco (*N. benthamiana*, 4 weeks old) leaves by *Agrobacterium* infiltration. After infection for 2 days, 1 mM precooled luciferin was sprayed on to the leaves, and then the samples were incubated in the dark for 5–10 min. The images were captured using a cooled charge‐coupled device imaging apparatus.

### Co‐IP assay

Co‐IP assay was performed as previously described with some modifications (Hu *et al*., 2019). Briefly, total protein was extracted from infiltrated tobacco (*N. benthamiana*, 4 weeks old) leaves (co‐expressing OsUbc13‐EGFP and OsSnRK1a) with protein extraction buffer (50 mM Tris–HCl (pH 7.5), 100 mM NaCl, 1 mM EDTA, 10 mM NaF, 5 mM Na_3_VO_4_, 0.25% Triton X‐100, 0.25% NP‐40, 1 mM PMSF, 1× protease inhibitor cocktail), and then incubated with 20 μL anti‐GFP agarose beads (Chromotek, gta‐20) for 2 h at 4 °C. The beads were washed five times with wash buffer (50 mM Tris–HCl (pH 7.5), 100 mM NaCl, 1 mM EDTA, 1 mM PMSF and 1× protease inhibitor cocktail), and the precipitated proteins were eluted with 2× SDS loading buffer at 95 °C for 3 min. The samples were subjected to immunoblot analysis using anti‐GFP (TransGene Biotech) and anti‐OsSnRK1a (PhytoAB) antibodies. For all immunoblot analyses for OsUbc13‐EGFP, the protein samples were not boiled.

### Bimolecular fluorescence complementation assay

The BiFC assay was performed as previously described (Bracha‐Drori *et al*., 2004; Liu *et al*., 2021). To produce a fusion with either the N‐ or the C‐terminal fragment of YFP, OsUbc13, OsSnRK1a, OsHRLI, OsSnRK1a^1–455^ and OsOTUB1.1 were subcloned into the pCAMBIA1300‐nYFP or pCAMBIA1300‐cYFP vectors, respectively. Corresponding BiFC plasmids and negative controls were co‐expressed in wild‐type tobacco (*N. benthamiana*, 4 weeks old) leaves by *Agrobacterium* infiltration. After infection for 2 days, the YFP fluorescence was detected with a 514 nm laser (Leica TCS SP5).

### Subcellular localization analysis

Subcellular localization assay was performed as described previously (Liu *et al*., 2021). Briefly, the full‐length CDS of *OsSnRK1a* was amplified by PCR and directionally inserted into pCAMBIA1300‐35S:EGFP. Cultures of the *A. tumefaciens* strain EHA105 harbouring the pCAMBIA1300‐35S:OsSnRK1a‐EGFP constructs were used to infect the healthy leaves of tobacco (*N. benthamiana*, 4 weeks old) expressing 35S:RFP‐histone 2B (nuclear marker, Martin *et al*., 2009). After infection for 2 days, the fluorescent signals were observed using a confocal microscope (Leica TCS SP5).

### Cell‐free protein synthesis and pull‐down assay

Coupled cell‐free transcription and translation reactions were performed as previously described (Zhang *et al*., 2020b) with some modifications. To generate plasmids for CFPS reactions, the full‐length CDSs of OsUbc13 and OsSnRK1a were inserted into pD2P‐8 × His‐GFP and pD2P‐3 × Flag‐GFP vectors by seamless cloning, respectively. Standard CFPS reactions were carried out in 1.5‐mL microcentrifuge tubes. Each reaction (15 μL) contains the following components: 16.7 μg/mL recombinant plasmids (8 × His‐OsUbc13‐GFP or 3 × Flag‐OsSnRK1a‐GFP), 25 mM HEPES‐KOH (pH 7.4), 120 mM potassium glutamate, 6 mM magnesium glutamate, 1.5 mM of each ATP, GTP, CTP, and UTP, 0.1 mM of each of 20 amino acids, 25 mM creatine phosphate, 1.7 mM DTT, 1 mM putrescine, 0.5 mM spermidine, 0.27 mg/mL creatine phosphokinase (from rabbit muscle; Sigma–Aldrich), 60 U T7 RNA polymerase (Thermo Fisher Scientific), and 50% (v/v) cell extract. All reactions were mixed using the above conditions and incubated at 23 °C for 5 h unless otherwise noted. The recombinant protein was purified with magnetic beads containing His and Flag tags, respectively.

Cell‐free expressed and purified OsUbc13 and OsSnRK1a fusion proteins (100 μL each) were mixed into the same 1.5‐mL centrifuge tube and incubated at 4 °C with rotation for 4 h. After that, 70 μL His‐tag magnetic beads were added and mixed. After incubation at 4 °C for 1 h, the mixture was placed on a magnetic stand, and the supernatant was discarded. 1 mL of washing buffer was added to the above 1.5‐mL centrifuge tube, mixed by inversion, centrifuged briefly and placed on a magnetic stand. When the solution was clear, the supernatant was discarded, and the process was repeated six times. Finally, the remaining magnetic beads in the centrifuge tube were mixed with 50 μL of loading buffer and heated at 100 °C for 5–10 min for immunoblotting analysis using anti‐His and anti‐Flag antibodies (TransGene Biotech).

### Detection of the protein levels of OsSnRK1a

The seedlings at 30‐day post‐sowing in soil were used to detect the protein levels of OsSnRK1a. Total protein was extracted with extraction buffer (50 mM Tris–HCl (pH 7.5), 100 mM NaCl, 1 mM EDTA, 10 mM NaF, 5 mM Na_3_VO_4_, 0.25% Triton X‐100, 0.25% NP‐40, 1 mM PMSF, 1× protease inhibitor cocktail) and adjusted to equal concentrations. OsSnRK1a abundance was detected by western blotting using the anti‐OsSnRK1a antibody (PhytoAB).

### SnRK1 activity assay

The rice seedlings at 30‐day post‐sowing in soil, or the tobacco leaves after transient expression of EGFP and OsSnRK1a‐EGFP for 2 days, were used to detect SnRK1 activity as previously described (Liang *et al*., 2021). Fresh plant samples (1 g) were ground in 1 mL of cold extraction buffer containing 100 mM Tricine‐NaOH (pH 8.0), 25 mM NaF, 5 mM dithiothreitol, 2 mM tetrasodium pyrophosphate, 0.5 mM ethylene diamine tetraacetic acid, 0.5 mM ethylene glycol tetraacetic acid, 1 mM benzamidine, 1 mM phenylmethylsulfonyl fluoride, 1 mM protease inhibitor cocktail (Sigma P9599), phosphatase inhibitors (PhosStop; Roche), and insoluble polyvinylpyrrolidone with a final concentration of 2% (w/v). The homogenate was transferred to two cold microfuge tubes and centrifuged at 12000 g for 5 min at 4 °C. A pre‐equilibrated 2.5‐mL centrifuge column was used to desalt the supernatant (750 μL). AMARA polypeptide was used as the substrate. SnRKl activity was measured using the Universal Kinase Activity Kit (R&D Systems, Zhang *et al*., 2009).

### ABA sensitivity analysis

To test ABA sensitivity, the wild‐type DJ and *OsUbc13*‐RNAi lines were germinated on 1/2 MS medium for 3 d. After germination, the seedlings with similar shoot and root length were transplanted to transparent plastic plates with 1/2 MS medium containing 3 μM of ABA or water as a control. After 1 week of growth, the phenotypes were recorded, and the shoot length of these seedlings was measured.

### Detection of the ubiquitination levels of OsSnRK1a

The rice seedlings at 30‐day post‐sowing in soil were used to detect the ubiquitination levels of OsSnRK1a as previously described (Chen *et al*., 2021). Total protein was extracted with extraction buffer (50 mM Tris–HCl (pH 7.5), 100 mM NaCl, 1 mM EDTA, 10 mM NaF, 5 mM Na_3_VO_4_, 0.25% Triton X‐100, 0.25% NP‐40, 1 mM PMSF, 1× protease inhibitor cocktail). Anti‐OsSnRK1a antibodies coupled to IgG agarose were added to the crude protein, incubated at 4 °C for 2 h, and washed at least five times with PBS. The obtained immunoprecipitates were suspended in protein loading buffer and boiled at 95 °C for 5 min before being analysed by SDS‐PAGE, and the separated proteins were transferred to a nitrocellulose membrane (GE Healthcare). The OsSnRK1a protein was then detected by probing the membrane with an anti‐OsSnRK1a antibody (PhytoAB), and polyubiquitinated forms were detected by probing with antibodies that recognize total ubiquitin conjugates, antibodies that specifically recognize K48‐polyubiquitin conjugates, or antibodies that specifically recognize K63‐polyubiquitin conjugates (Abcam).

## Funding

This study was supported by the National Natural Science Foundation of China (grant nos. 32171932 and 31601232), the Natural Science Foundation of Fujian Province (grant no. 2021 J01088), the Joint Funds of Science and Technology R&D Program of Henan Province (grant no. 222301420101), Fujian Agriculture and Forestry University Program for Distinguished Young Scholar (grant no. xjq201706), and China Postdoctoral Science Foundation (grant no. 2017 M612108).

## Author contributions

J.L. and W.X. designed the project and wrote the manuscript. J.L., B.N., and B.Y. conducted the experiments, organized, and analysed the data. F.X., Q.Z., and Y.W. contributed to the experimental design and manuscript revision. All authors have read and approved the final manuscript.

## Conflict of interest statement

None declared.

## Supporting information


**Figure S1** Several agronomic traits of DJ and *OsUbc13*‐RNAi plants. (a) Plant height. (b) Tiller number per plant. (c) Grain number per panicle. (D) 1000‐grain weight. Data are shown as means ±SE; (a) to (c), *n* = 17; (d), *n* = 13 (***P < 0.001; Student's *t*‐test). All phenotypic data were measured in paddy‐grown rice plants under normal cultivation conditions.
**Figure S2** MDA and Photosynthetic pigment content in DJ and *OsUbc13*‐RNAi leaves. The rice leaves at 30‐day post sowing in soil were used to measure MDA (a) and photosynthetic pigment (b). Data are shown as means ±SE; *n* = 3 (*P < 0.05, ***P < 0.01, ***P < 0.001; Student's *t*‐test).
**Figure S3** Overexpression of *OsUbc13* did not affect the resistance to *M. oryzae*. (a) qRT‐PCR analysis of *OsUbc13* expression in *OsUbc13*‐OE lines (OE17‐2 and OE18‐3). *OsActin1* gene was used as an internal control. Data are shown as means ±SE; *n* = 3 (***P < 0.001; Student's *t*‐test). (b) The lesions on DJ and *OsUbc13*‐OE leaves at 8‐day after punch inoculation with the compatible *M. Oryzae* isolate GUY11. Scale bar = 1 cm. (c) Relative lesion area (%) in leaves of (b) indicates no significant differences between DJ and *OsUbc13*‐OE. Data are shown as means ±SE; *n* = 6.
**Figure S4** Protein sequences of rice OsUbc13 and tomato Fni3. Red letters indicate functionally important amino acid residues for biochemical activities of Ubc13. Cys‐89, the active site for ubiquitin thioester formation; Met‐66, which is involved in the interaction with an E3 ligase; Glu‐57, Phe‐59, and Arg‐72, three pocket residues, determine binding specificity for Uev protein. Purple letters indicate the only 5 different amino acid residues between OsUbc13 and Fni3.
**Figure S5** Mutation of *OsUEV1B* or *OsVDAC1* did not affect the resistance to *M. oryzae*. (A) The lesions on DJ, *osuev1b*, and *osvdac1* leaves at 8‐day after punch inoculation with the compatible *M. Oryzae* isolate GUY11. Scale bar = 1 cm. (B) Relative lesion area (%) in leaves of (A) indicates no significant differences between DJ and *osuev1b* or *osvdac1*. Data are shown as means ±SE; *n* = 6.
**Figure S6** SnRK1 kinase activity in tobacco leaves after transient expression of empty EGFP or OsSnRK1a‐EGFP fusion protein. (a) qRT‐PCR analysis of *OsSnRK1a* expression in tobacco leaves after infection for 2 days. *NtEF‐1α* gene was used as an internal control. Data are shown as means ±SE; *n* = 3 (***P < 0.001; Student's *t*‐test). (b) SnRK1 activity in tobacco leaves after infection for 2 days. Data are shown as means ±SE; *n* = 3 (***P < 0.001; Student's *t*‐test).
**Figure S7** qRT‐PCR analysis of *OsSnRK1a* expression level in transgenic lines of *OsUbc13*. (a) *OsUbc13*‐RNAi lines. (b) *OsUbc13*‐OE lines. Data are shown as means ±SE; *n* = 3.
**Figure S8** The *OsUbc13*‐RNAi lines exhibited increased ABA sensitivity. (a) Phenotypes of DJ and *OsUbc13*‐RNAi seeds after 8 days of growth on 1/2 MS medium with or without 3 μM ABA. Scale bar = 2 or 5 cm. (b) Shoot and root lengths with ABA treatment. Data are shown as means ±SE; *n* = 10 (**P < 0.01, ***P < 0.001; Student's *t*‐test). (c) Shoot and root lengths without ABA treatment. Data are shown as means ±SE; *n* = 10 (**P < 0.01; Student's *t*‐test).
**Figure S9** Constitutive expression of several defense‐related genes in *OsOTUB1.1*‐OE and wild‐type ZH11. Total RNA was extracted from the leaves of *OsOTUB1.1*‐OE and ZH11 plants at 30‐day post sowing in soil. qRT‐PCR was used to analyze the genes expression. (a) *OsOTUB1.1* expression. (b) to (f) The expression of defense‐related genes involved in JA signaling/synthetic pathway. (g) to (m) The expression of defense‐related genes involved in SA signaling pathway. (n) *OsSnRK1a* expression. Data are shown as means ±SE; *n* = 3 (**P < 0.01, ***P < 0.001; Student's *t*‐test).
**Table S1** Primers used in this study
