# Supplementary material for: Rice ubiquitin‐conjugating enzyme OsUbc13 negatively regulates immunity against pathogens by enhancing the activity of OsSnRK1a
Source: Plant Biotechnol J. 2023 Apr 27;21(8):1590–610. doi: 10.1111/pbi.14059 (PMC10363768; doi:10.1111/pbi.14059)
Supplement: Supplementary file 1 — Figure S1 Several agronomic traits of DJ and OsUbc13‐RNAi plants. (a) Plant height. (b) Tiller number per plant. (c) Grain number per panicle. (D) 1000‐grain weight. Data are shown as means ±SE; (a) to (c), n = 17; (d), n = 13 (***P < 0.001; Student's t‐test). All phenotypic data were measured in paddy‐grown rice plants under normal cultivation conditions. Figure S2 MDA and Photosynthetic pigment content in DJ and OsUbc13‐RNAi leaves. The rice leaves at 30‐day post sowing in soil were used to measure MDA (a) and photosynthetic pigment (b). Data are shown as means ±SE; n = 3 (*P < 0.05, ***P < 0.01, ***P < 0.001; Student's t‐test). Figure S3 Overexpression of OsUbc13 did not affect the resistance to M. oryzae. (a) qRT‐PCR analysis of OsUbc13 expression in OsUbc13‐OE lines (OE17‐2 and OE18‐3). OsActin1 gene was used as an internal control. Data are shown as means ±SE; n = 3 (***P < 0.001; Student's t‐test). (b) The lesions on DJ and OsUbc13‐OE leaves at 8‐day after punch inoculation with the compatible M. Oryzae isolate GUY11. Scale bar = 1 cm. (c) Relative lesion area (%) in leaves of (b) indicates no significant differences between DJ and OsUbc13‐OE. Data are shown as means ±SE; n = 6. Figure S4 Protein sequences of rice OsUbc13 and tomato Fni3. Red letters indicate functionally important amino acid residues for biochemical activities of Ubc13. Cys‐89, the active site for ubiquitin thioester formation; Met‐66, which is involved in the interaction with an E3 ligase; Glu‐57, Phe‐59, and Arg‐72, three pocket residues, determine binding specificity for Uev protein. Purple letters indicate the only 5 different amino acid residues between OsUbc13 and Fni3. Figure S5 Mutation of OsUEV1B or OsVDAC1 did not affect the resistance to M. oryzae. (A) The lesions on DJ, osuev1b, and osvdac1 leaves at 8‐day after punch inoculation with the compatible M. Oryzae isolate GUY11. Scale bar = 1 cm. (B) Relative lesion area (%) in leaves of (A) indicates no significant differences betwee [file PBI-21-1590-s001.docx]

**Supplemental Data**


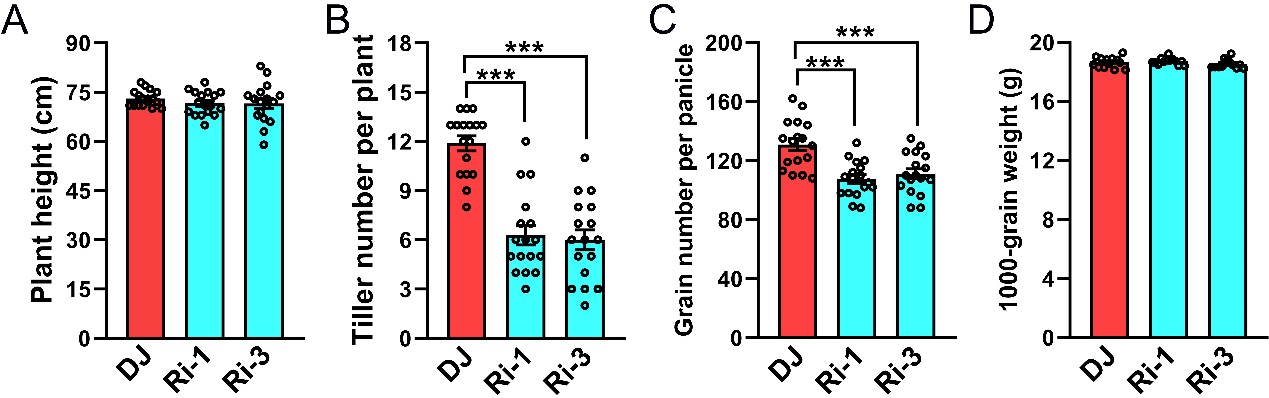


**Supplemental Figure S1.** Several agronomic traits of DJ and *OsUbc13*-RNAi plants. (A) Plant height. (B) Tiller number per plant. (C) Grain number per panicle. (D) 1000-grain weight. Data are shown as means ±SE; (A) to (C), *n* = 17; (D), *n* = 13 (****P* < 0.001; Student’s *t*-test). All phenotypic data were measured in paddy-grown rice plants under normal cultivation conditions.


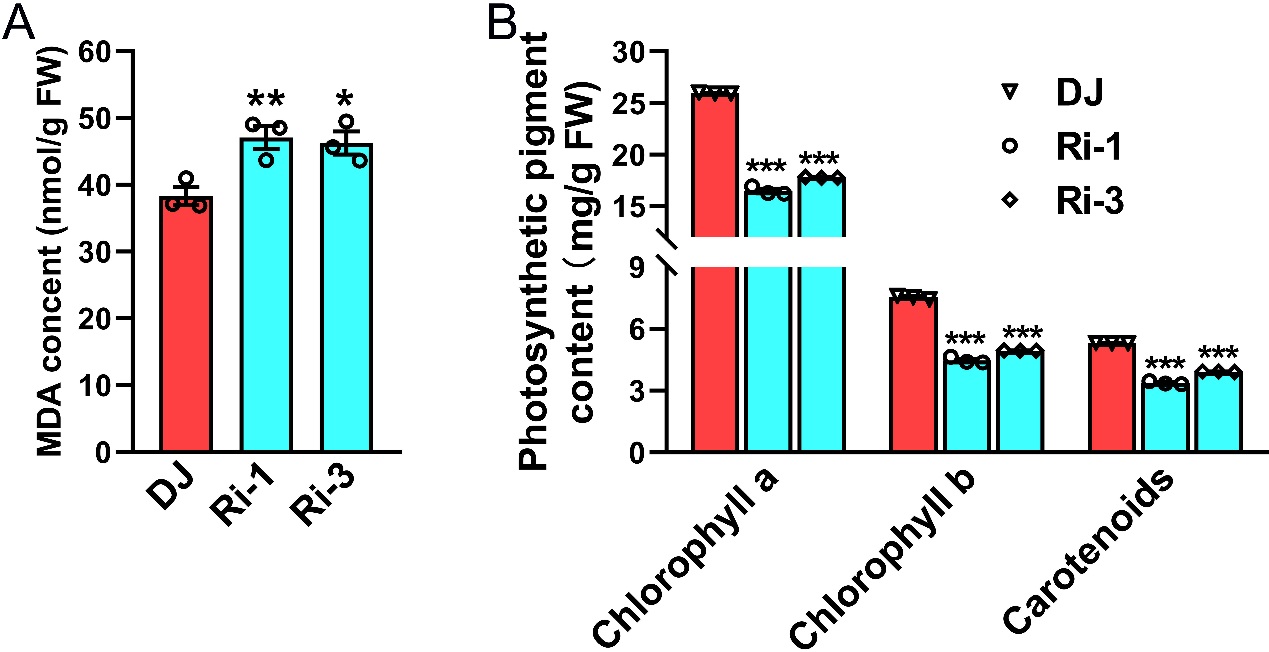


**Supplemental Figure S2.** MDA and Photosynthetic pigment content in DJ and *OsUbc13*-RNAi leaves. The rice leaves at 30-day post sowing in soil were used to measure MDA (A) and photosynthetic pigment (B). Data are shown as means ±SE; *n* = 3 (**P* < 0.05, ****P* < 0.01, ****P* < 0.001; Student’s *t*-test).


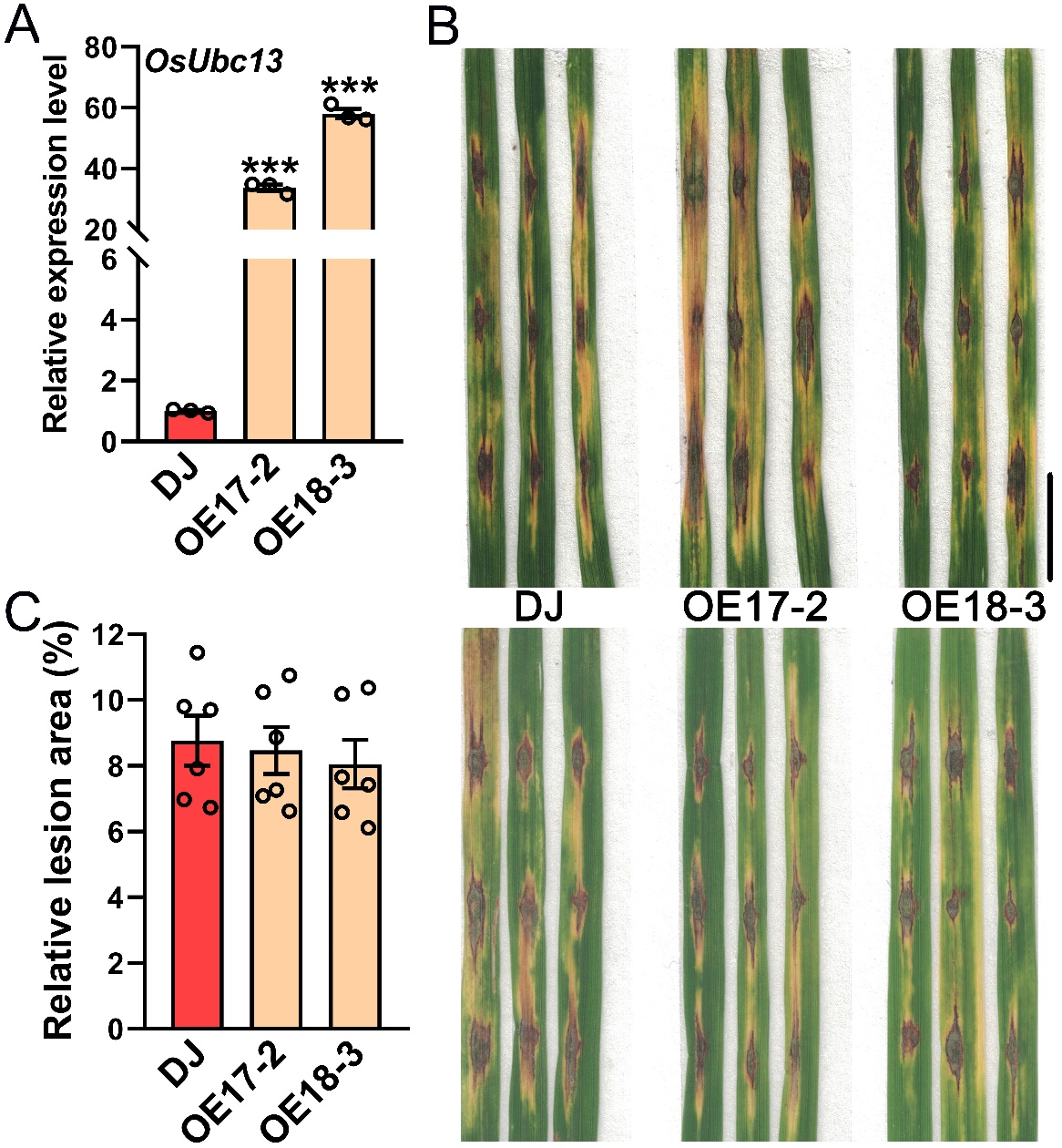


**Supplemental Figure S3.** Overexpression of *OsUbc13* did not affect the resistance to *M. oryzae*. (A) qRT-PCR analysis of *OsUbc13* expression in *OsUbc13*-OE lines (OE17-2 and OE18-3). *OsActin1* gene was used as an internal control. Data are shown as means ±SE; *n* = 3 (****P* < 0.001; Student’s *t*-test). (B) The lesions on DJ and *OsUbc13*-OE leaves at 8-day after punch inoculation with the compatible *M. Oryzae* isolate GUY11. Scale bar = 1 cm. (C) Relative lesion area (%) in leaves of (B) indicates no significant differences between DJ and *OsUbc13*-OE. Data are shown as means ±SE; *n* = 6.


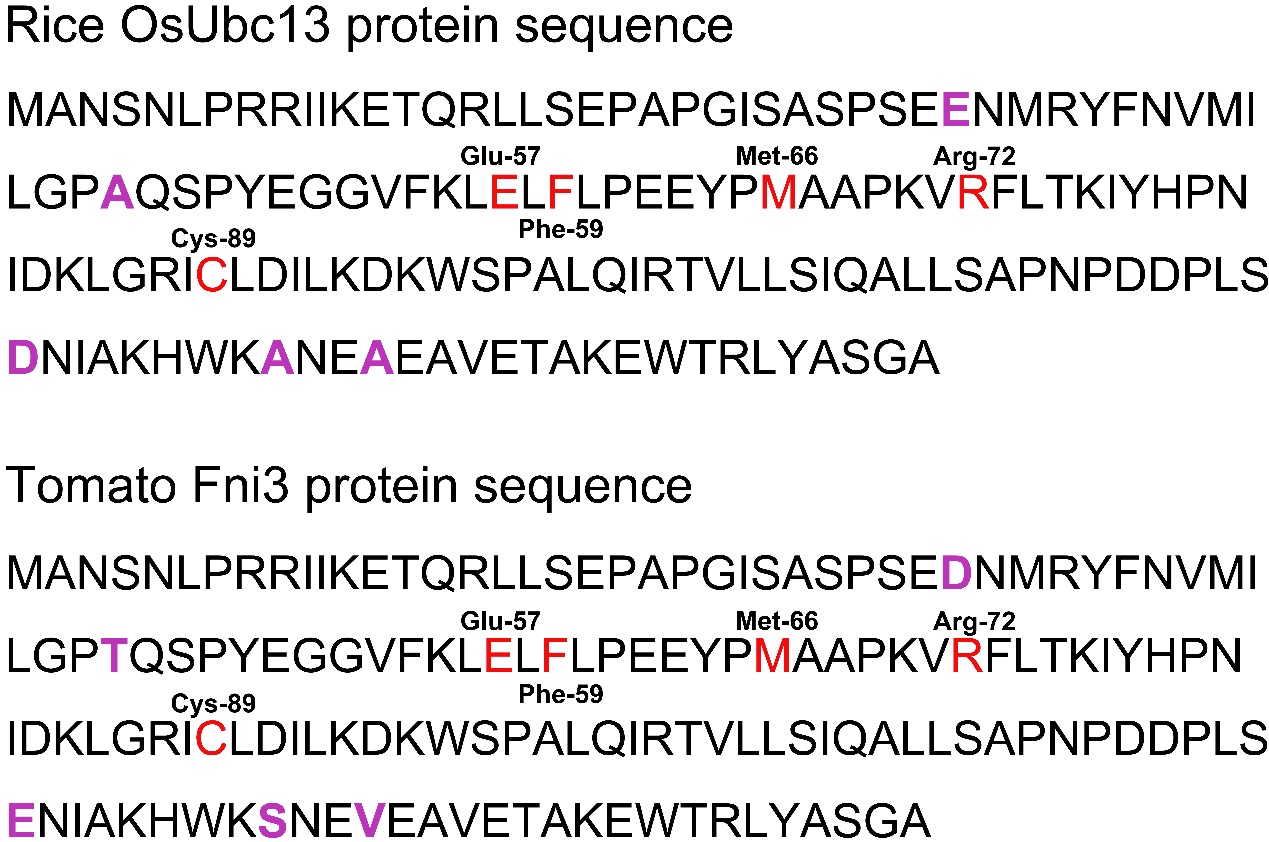


**Supplemental Figure S4.** Protein sequences of rice OsUbc13 and tomato Fni3. Red letters indicate functionally important amino acid residues for biochemical activities of Ubc13. Cys-89, the active site for ubiquitin thioester formation; Met-66, which is involved in the interaction with an E3 ligase; Glu-57, Phe-59, and Arg-72, three pocket residues, determine binding specificity for Uev protein. Purple letters indicate the only 5 different amino acid residues between OsUbc13 and Fni3.


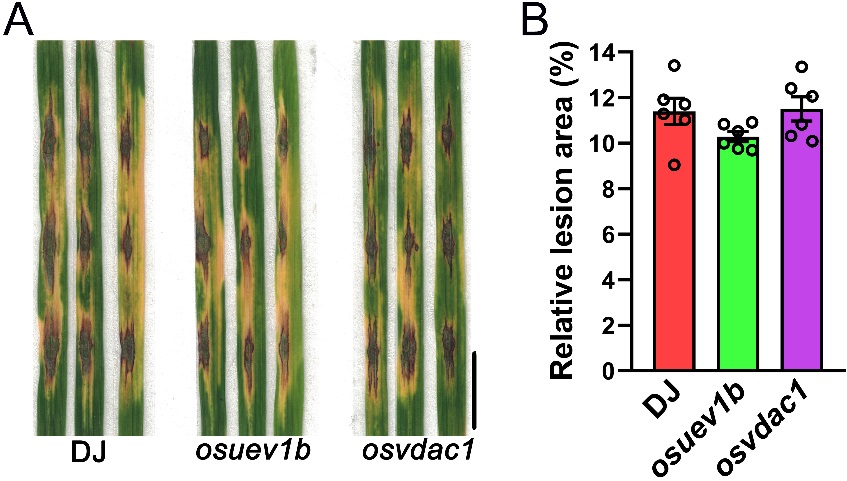


**Supplemental Figure S5.** Mutation of *OsUEV1B* or *OsVDAC1* did not affect the resistance to *M. oryzae*. (A) The lesions on DJ, *osuev1b*, and *osvdac1* leaves at 8-day after punch inoculation with the compatible *M. Oryzae* isolate GUY11. Scale bar = 1 cm. (B) Relative lesion area (%) in leaves of (A) indicates no significant differences between DJ and *osuev1b* or *osvdac1*. Data are shown as means ±SE; *n* = 6.


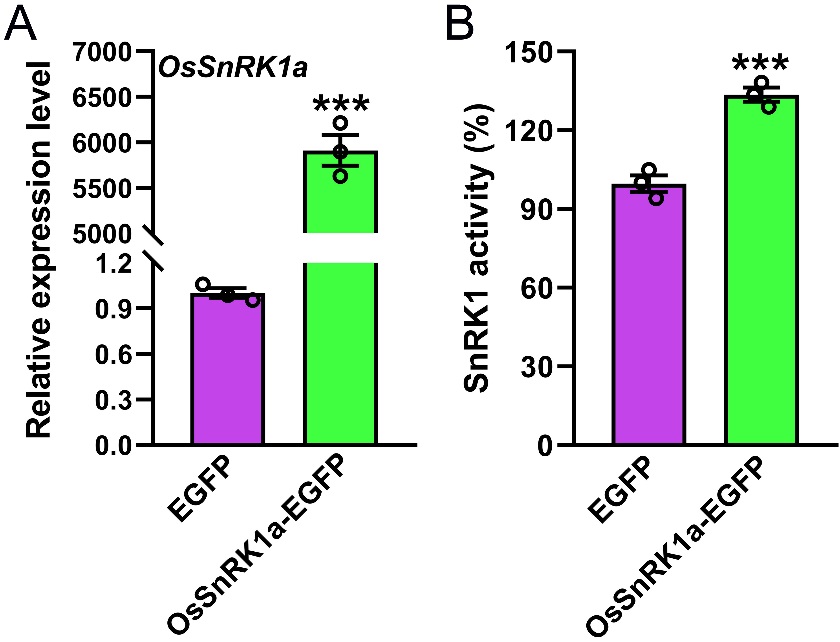


**Supplemental Figure S6.** SnRK1 kinase activity in tobacco leaves after transient expression of empty EGFP or OsSnRK1a-EGFP fusion protein. (A) qRT-PCR analysis of *OsSnRK1a* expression in tobacco leaves after infection for 2 days. *NtEF-1α* gene was used as an internal control. Data are shown as means ±SE; *n* = 3 (****P* < 0.001; Student’s *t*-test). (B) SnRK1 activity in tobacco leaves after infection for 2 days. Data are shown as means ±SE; *n* = 3 (****P* < 0.001; Student’s *t*-test).


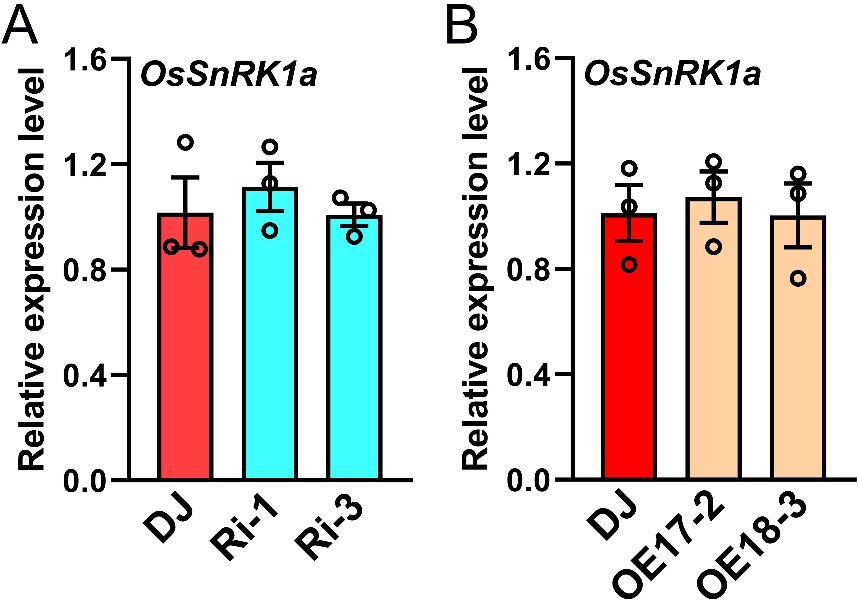


**Supplemental Figure S7.** qRT-PCR analysis of *OsSnRK1a* expression level in transgenic lines of *OsUbc13*. (A) *OsUbc13*-RNAi lines. (B) *OsUbc13*-OE lines. Data are shown as means ±SE; *n* = 3.


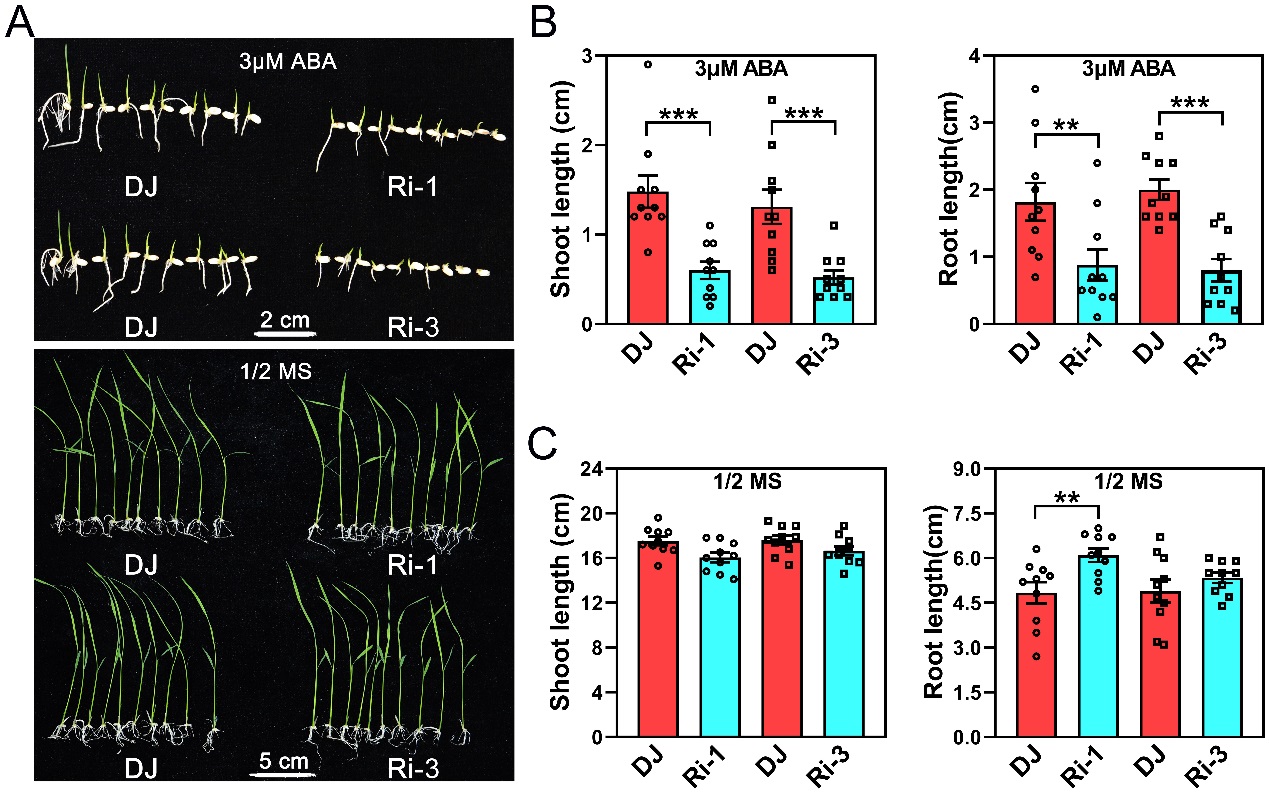


**Supplemental Figure S8.** The *OsUbc13*-RNAi lines exhibited increased ABA sensitivity. (A) Phenotypes of DJ and *OsUbc13*-RNAi seeds after 8 days of growth on 1/2 MS medium with or without 3 μM ABA. Scale bar = 2 or 5 cm. (B) Shoot and root lengths with ABA treatment. Data are shown as means ±SE; *n* = 10 (***P* < 0.01, ****P* < 0.001; Student’s *t*-test). (C) Shoot and root lengths without ABA treatment. Data are shown as means ±SE; *n* = 10 (***P* < 0.01; Student’s *t*-test).


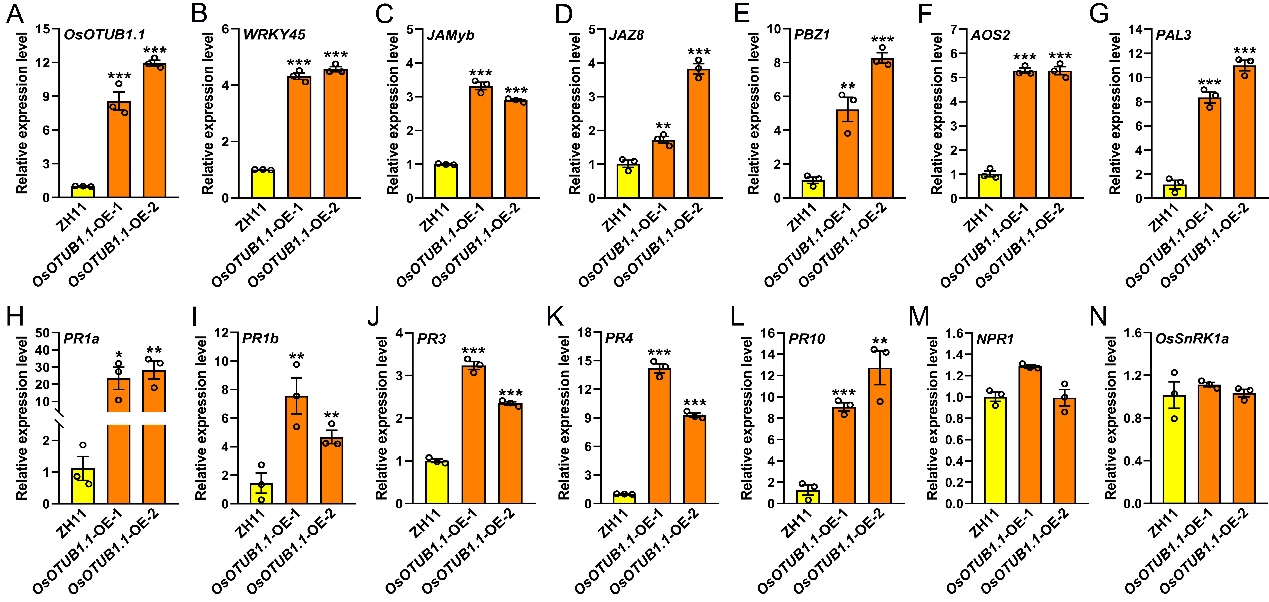


**Supplemental Figure S9.** Constitutive expression of several defense-related genes in *OsOTUB1.1*-OE and wild-type ZH11. Total RNA was extracted from the leaves of *OsOTUB1.1*-OE and ZH11 plants at 30-day post sowing in soil. qRT-PCR was used to analyze the genes expression. (A) *OsOTUB1.1* expression. (B) to (F) The expression of defense-related genes involved in JA signaling/synthetic pathway. (G) to (M) The expression of defense-related genes involved in SA signaling pathway. (N) *OsSnRK1a* expression. Data are shown as means ±SE; *n* = 3 (***P* < 0.01, ****P* < 0.001; Student’s *t*-test).

**Supplemental Table S1.** Primers used in this study.

**Table S1 Primers used in this study**

| **Primer name** | **Primer sequence (5′→3′)** |
| --- | --- |
| **For RNAi and over-expressing constructs** | |
| *OsUbc13*-RNAi F | ATAGGTACCACTAGTGCGAATCATCAAGGAGACGCAG |
| *OsUbc13*-RNAi R | ATAGGATCCGAGCTCCAGGAACCTAACCTTTGGAGCAG |
| *OsUbc13*-OE F | ATACTGCAGATGGCCAACAGCAACCTCCCCCG |
| *OsUbc13*-OE R | ATACCCGGGTTATGCACCGCTGGCATACAGGCGA |
| *OsSnRK1a*-RNAi F ATAGGTACCACTAGTACCGGATTGGCAAAACCCTA | |
| *OsSnRK1a*-RNAi R ATAGGATCCGAGCTCCCTCATAAAGGCGAATGATATGTG | |
|  | |
| **For BiFC assay constructs** | |
| OsUbc13-nYFP F | AAGAGACAGGATCCGAATTCATGGCCAACAGCAACCTCC |
| OsUbc13-nYFP R | ACCTCCACTAGTGTCGACTGCACCGCTGGCATACAG |
| OsUbc13-cYFP F | CTGATCAAGAGACAGGATCCATGGCCAACAGCAACCTCC |
| OsUbc13-cYFP R | CCACCTCCACTAGTGTCGACTGCACCGCTGGCATACAG |
| OsSnRK1a-nYFP F | TCTGATCAAGAGACAGGATCCATGGAGGGAGCTGGCAGAGA |
| OsSnRK1a-nYFP R | CATACCTCCACTAGTGTCGACAAGGACTCTCAGCTGAGTTA |
| OsSnRK1a-cYFP F | CTGATCAAGAGACAGGATCCATGGAGGGAGCTGGCAGAGA |
| OsSnRK1a-cYFP R | CCACCTCCACTAGTGTCGACAAGGACTCTCAGCTGAGTTA |
| OsSnRK1a**^1-455^**-nYFP F | AAGAGACAGGATCCGAATTCATGGAGGGAGCTGGCAGAGA |
| OsSnRK1a**^1-455^**-nYFP R | ACCTCCACTAGTGTCGACGTCATCAGTTTCAATTA |
| OsSnRK1a**^1-455^**-cYFP F | CTGATCAAGAGACAGGATCCATGGAGGGAGCTGGCAGAGA |
| OsSnRK1a**^1-455^**-cYFP R | CCACCTCCACTAGTGTCGACGTCATCAGTTTCAATTA |
| OsHRLI-nYFP F | AAGAGACAGGATCCGAATTCATGGGCTTCCTGTCCTTCGCCG |
| OsHRLI-nYFP R | ACCTCCACTAGTGTCGACGGTCGACTTTGGTTTAGGGGCCT |
| OsHRLI-cYFP F | CTGATCAAGAGACAGGATCCATGGGCTTCCTGTCCTTCGCCG |
| OsHRLI-cYFP R | CCACCTCCACTAGTGTCGACGGTCGACTTTGGTTTAGGGGCCT |
| OsOTUB1.1-nYFP F | AAGAGACAGGATCCGAATTCATGGGCGGGGACTACTACCA |
| OsOTUB1.1-nYFP R | ACCTCCACTAGTGTCGACCTTCGGGTAGAGAATGTCG |
| OsOTUB1.1-cYFP F | CTGATCAAGAGACAGGATCCATGGGCGGGGACTACTACCA |
| OsOTUB1.1-cYFP R | CCACCTCCACTAGTGTCGACCTTCGGGTAGAGAATGTCG |
|  |  |
| **For LCI assay constructs** | |
| cLuc-OsUbc13 F | GGGGTACCATGGCCAACAGCAACCT |
| cLuc-OsUbc13 R | GCGTCGACTTATGCACCGCTGGCAT |
| OsSnRK1a-nLuc F | CGGGGGACGAGCTCGGTACCATGGAGGGAGCTGGCAGAGA |
| OsSnRK1a-nLuc R | GCGTACGAGATCTGGTCGACAAGGACTCTCAGCTGAGTTA |
| cLuc-OsOTUB1.1 F | GGGGTACCATGGGCGGGGACTACTACCA |
| cLuc-OsOTUB1.1 R | GCGTCGACTCACTTCGGGTAGAGAATGTCG |
|  | |
| **For Co-IP and subcellular localization constructs** | |
| OsUbc13-EGFP F | GGACTAGTATGGAGCATGCTACCTGTGATGA |
| OsUbc13-EGFP R | GGGGTACCTACAGTGAATCTCGAGCTAGCTATACCT |
| OsSnRK1a-EGFP F | ATAGGATCCATGGAGGGAGCTGGCAGAGA |
| OsSnRK1a-EGFP R | ATAGTCGACAAGGACTCTCAGCTGAGTTA |
|  |  |
| **For pull-down assay constructs** | |
| 3×Flag-SnRK1a-GFP F | GACAAGGGTTCTGGAGGATCCATGGAGGGAGCTGGCAGAGA |
| 3×Flag-SnRK1a-GFP R | GCTTCCTCCATGGCTGGATCCAAGGACTCTCAGCTGAGTTAGAAAGG |
| 8×His-OsUbc13-GFP F | CATCACGGGAGCGGCGGATCCATGGCCAACAGCAACCTCC |
| 8×His-OsUbc13-GFP R | GCTTCCTCCATGGCTGGATCCTGCACCGCTGGCATACAGG |
|  |  |
| **For yeast two-hybrid assay constructs** | |
| BK-OsUbc13 F | GGAATTCATGGCCAACAGCAACCTCCCCCG |
| BK-OsUbc13 R | AACTGCAGTTATGCACCGCTGGCATACAGGCGA |
| AD-OsHRLI F | CGGAATTCATGGGCTTCCTGTCCTTCGCCGGGA |
| AD-OsHRLI R | CGAGCTCTTAGGTCGACTTTGGTTTAGGGGCCT |
| AD-OsCPI F | CGGAATTCATGCTTCGCCGCCGCGGCTT |
| AD-OsCPI R | CGAGCTCTCACACCGAGTATGTCTGAG |
| AD-OsYchF1 F | CGGAATTCATGCCGCCCAAGGCGTCCAAGAAGG |
| AD-OsYchF1 R | CGAGCTCTCACTTCTTTCCACCTCCAGACACG |
| AD-OsSnRK1a F | CGGAATTCATGGAGGGAGCTGGCAGAGATGGGA |
| AD-OsSnRK1a R | CGAGCTCTTAAAGGACTCTCAGCTGAGTTAGAAAGGC |
| AD-OsSnRK1a**^1-455^** F | CGGAATTCATGGAGGGAGCTGGCAGAGA |
| AD-OsSnRK1a**^1-455^** R | CGAGCTCTTAGTCATCAGTTTCAATTA |
| AD-OsSnRK1a**^1-327^** F | CGGAATTCATGGAGGGAGCTGGCAGAGA |
| AD-OsSnRK1a**^1-327^** R | CGAGCTCTTAGTCCAATAGTAAATAGT |
| AD-OsSnRK1a**^1-286^** F | CGGAATTCATGGAGGGAGCTGGCAGAGA |
| AD-OsSnRK1a**^1-286^** R | CGAGCTCTTATTTAACCTGTTGTGCAG |
| AD-OsSnRK1a**^287-505^** F | CGGAATTCAAGCTCGACGATGAAACTCTGA |
| AD-OsSnRK1a**^287-505^** R | CGAGCTCTTAAAGGACTCTCAGCTGAG |
| AD-OsSnRK1a**^328-505^** F | CGGAATTCAATAGGCTGCGCACAACCA |
| AD-OsSnRK1a**^328-505^** R | CGAGCTCTTAAAGGACTCTCAGCTGAG |
| AD-OsSnRK1a**^456-505^** F | CGGAATTCATGAGTGAGAAATCAACCCA |
| AD-OsSnRK1a**^456-505^** R | CGAGCTCTTAAAGGACTCTCAGCTGAG |
| BK-OsUbc13**^C89G^** F | GAGAATGTCAAGGCCTATCCTACCAAGCTTGTCAATGTTGG |
| BK-OsUbc13**^C89G^** R | CCAACATTGACAAGCTTGGTAGGATAGGCCTTGACATTCTC |
| AD-OsSnRK1a**^K43M^** F | ACGGCGATTGAGGATCATGATTGCCACCTTGTG |
| AD-OsSnRK1a**^K43M^** R | CACAAGGTGGCAATCATGATCCTCAATCGCCGT |
| AD-OsSnRK1a**^K139R^** F | AGGTTCTCTGGCCTAAGATCACGATGAACCACCATG |
| AD-OsSnRK1a**^K139R^** R | CATGGTGGTTCATCGTGATCTTAGGCCAGAGAACCT |
| BK-OsOTUB1.1 F | CGGAATTCATGGGCGGGGACTACTACCA |
| BK-OsOTUB1.1 R | CGGGATCCTCACTTCGGGTAGAGAATGTCG |
|  |  |
| **For qRT-PCR assay** |  |
| q*OsUbc13* F | GCTTGAACTCTTTTTACCTGAGGA |
| q*OsUbc13* R | GGGTGGTATATTTTGGTCAGGA |
| q*OsSnRK1a* F | GCAAAACGAGGCGACAGT |
| q*OsSnRK1a* R | GCTGGAGTTACTTGAGCGAGA |
| q*OsOTUB1.1* F | AAATCCAAAGGAGGGCCGAG |
| q*OsOTUB1.1* R | CCAACGCATCTGATAGGGCA |
| q*ICS1* F | TATGGTGCTATCCGCTTCGAT |
| q*ICS1* R | CGAGAACCGAGCTCTCTTCAA |
| q*AOS2* F | CTCGTCGGAAGGCTGTTGCT |
| q*AOS2* R | ACGATTGACGGCGGAGGTT |
| q*JAMyb* F | CCGAGCATGGTGACTAGCTCATCTT |
| q*JAMyb* R | CCTTGCACCCAACCGTTAAGCTGTT |
| q*JAZ8* F | CCAAACACGGCGGAAACAG |
| q*JAZ8* R | GGTGGACGGGAAGTTCTCAAAG |
| q*PBZ1* F | CCCTGCCGAATACGCCTAA |
| q*PBZ1* R | CTCAAACGCCACGAGAATTTG |
| q*WRKY45* F | TTCCTTGTTGATGTGTCGTCTCA |
| q*WRKY45* R | CCCCCAGCTCATAATCAAGAAC |
| q*PR1a* F | GGAAGTACGGCGAGAACATC |
| q*PR1a* R | TGGTCGTACCACTGCTTCTC |
| q*PR1b* F | AGAACTACGCCAGCCAGAGAAG |
| q*PR1b* R | TTCTCGCCAAGGTTGTTCCG |
| q*PR3* F | CGTGTCTGTGGAGAGCGTGGTC |
| q*PR3* R | TCGTCGTTGGTGCGGTCATTGG |
| q*PR4* F | AGTATGGATGGACCGCCTTCTGT |
| q*PR4* R | CTCGCAATTATTGTCGCACCTGTTC |
| q*PR10* F | CACCATCTACACCATGAAGC |
| q*PR10* R | AGCACATCCGACTTTAGGAC |
| q*NPR1* F | GGCAGGTGAGAGTCTACGAGGAA |
| q*NPR1* R | GCTGTCATCCGAGCTAAGTGTT |
| q*PAL3* F | CGCTGAGGCGTTTAAGATTG |
| q*PAL3* R | GGCAAGGACAGCAAGAATG |
| q*Actin1* F | TGGCATCTCTCAGCACATTCC |
| q*Actin1* R | TGCACAATGGATGGGCCAGA |
| q*NtEF-1α* F | TGAGATGCACCACGAAGCTC |
| q*NtEF-1α* R | CCAACATTGTCACCAGGAAGTG |
|  |  |
| **For relative fungal biomass detection** | |
| q*OsUbq* F | ACGATTGATTTAACCAGTCCATGA |
| q*OsUbq* R | TTCTGGTCCTTCCACTTTCAG |
| q*MoPot2* F | ACGACCCGTCTTTACTTATTTGG |
| q*MoPot2* R | AAGTAGCGTTGGTTTTGTTGGAT |
|  |  |
| **For positive test of transgenic plants** | |
| HYG F | GCTGTTATGCGGCCATTGTC |
| HYG R | GACGTCTGTCGAGAAGTTTC |
| G148 F | GGAGCGGCGATACCGTAAAGCACGA |
| G148 R | GCTATGACTGGGCACAACAGACAAT |
|  |  |
